# Biogeochemical Typing of Paddy Field by a Data-Driven Approach Revealing Sub-Systems within a Complex Environment - A Pipeline to Filtrate, Organize and Frame Massive Dataset from Multi-Omics Analyses

**DOI:** 10.1371/journal.pone.0110723

**Published:** 2014-10-20

**Authors:** Diogo M. O. Ogawa, Shigeharu Moriya, Yuuri Tsuboi, Yasuhiro Date, Álvaro R. B. Prieto-da-Silva, Gandhi Rádis-Baptista, Tetsuo Yamane, Jun Kikuchi

**Affiliations:** 1 Biotechnology and Natural Resources Program, University of the State of the Amazonas, Manaus, AM, Brazil; 2 Laboratory of Biochemistry and Biotechnology, Institute for Marine Sciences, Federal University of Ceara, Fortaleza, CE, Brazil; 3 Center for Environment and Biodiversity Studies, University of the State of the Amazonas, Manaus, AM, Brazil; 4 RIKEN Center for Sustainable Resource Science, and Biomass Engineering Corporation Division, Yokohama, Japan; 5 RIKEN Antibiotics Laboratory, Yokohama, Japan; 6 Graduate School of Medical Life Science, Yokohama City University, Suehiro-cho, Tsurumi-ku, Yokohama, Japan; 7 Laboratory of Genetics, Butantan Institute, Sao Paulo, SP, Brazil; 8 Center of Biotechnology of Amazon, Manaus, AM, Brazil; 9 Graduate School of Bioagricultural Sciences, Nagoya University, Nagoya, Japan; Wilfrid Laurier University, Canada

## Abstract

We propose the technique of biogeochemical typing (BGC typing) as a novel methodology to set forth the sub-systems of organismal communities associated to the correlated chemical profiles working within a larger complex environment. Given the intricate characteristic of both organismal and chemical consortia inherent to the nature, many environmental studies employ the holistic approach of multi-omics analyses undermining as much information as possible. Due to the massive amount of data produced applying multi-omics analyses, the results are hard to visualize and to process. The BGC typing analysis is a pipeline built using integrative statistical analysis that can treat such huge datasets filtering, organizing and framing the information based on the strength of the various mutual trends of the organismal and chemical fluctuations occurring simultaneously in the environment. To test our technique of BGC typing, we choose a rich environment abounding in chemical nutrients and organismal diversity: the surficial freshwater from Japanese paddy fields and surrounding waters. To identify the community consortia profile we employed metagenomics as high throughput sequencing (HTS) for the fragments amplified from Archaea rRNA, universal 16S rRNA and 18S rRNA; to assess the elemental content we employed ionomics by inductively coupled plasma optical emission spectroscopy (ICP-OES); and for the organic chemical profile, metabolomics employing both Fourier transformed infrared (FT-IR) spectroscopy and proton nuclear magnetic resonance (^1^H-NMR) all these analyses comprised our multi-omics dataset. The similar trends between the community consortia against the chemical profiles were connected through correlation. The result was then filtered, organized and framed according to correlation strengths and peculiarities. The output gave us four BGC types displaying uniqueness in community and chemical distribution, diversity and richness. We conclude therefore that the BGC typing is a successful technique for elucidating the sub-systems of organismal communities with associated chemical profiles in complex ecosystems.

## Introduction

Unravelling trends that rule complex aquatic environments is a puzzling task due to the myriad of possibilities of interactions presented between and within the hosted organismal consortia with organic and inorganic compounds.

As explaining the totality of the interactions is a goal hard to achieve, if not plainly impossible considering the never ending development on science, therefore, here we intend to frame sub-systems co-existing within a larger system using a data-driven approach [1]. To comprehend such interactions we gave rise to the biogeochemical typing (BGC typing), a flexible tool to bring forth and individualize a subset of structures underlying in the studied environment based on the correlation between the community and chemical profiles analysed.

The BGC typing analysis is a pipeline built using integrative statistical analysis and can treat massive datasets as used here produced by multi-omics analysis [2] which would otherwise be hard to visualize [3] and process [4]. It filters, organizes and frames the data based on the strength of the mutual trends working within the environment.

The multi-omics analyses here was composed by metagenomics which gave the community consortia profile, ionomics showing the elemental content a metabolomics for the organic chemical profile. Here, we regard metagenomics as applying solely to the characterization of small-subunit ribosomal RNA. Therefore, the multi-omics analysis provided who is there and what is there as explained as following.

In this study we researched on the aquatic environment of three distinct paddy fields and surrounding water located in Saitama Prefecture, Japan. The paddy field is the source of one of the most important staple foods in the world and a rich environment comparable to a natural wetland: more than merely the ability to sustain crops, it harbours an intricate net of life, and it is able to support even higher-trophic level organisms such as fish [5].

In a complex environment, one can find thousands of different organisms thriving. To answer who is there, we performed the metagenomics to identify the organismal consortia. The identification was expressed as operational taxonomic units (OTUs) [6] retrieved by high throughput sequencing (HTS) the polymerase chain reaction (PCR) products of Archaea-specific and universal 16S (Archaeal genes excluded) and universal 18S small-subunit ribosomal RNA primers.

To assess what is there we joined the pieces of information from ionomics and metabolomics.

The ionomics is the elemental analysis evaluating its variation over a set of samples in an approach as the one applied to plant assay [7]. The ionomic analysis was assessed by the use of inductively coupled plasma optical emission spectroscopy (ICP-OES).

For the metabolomics we used two techniques, the attenuated total reflectance Fourier transformed infrared (FT-IR) and the proton nuclear magnetic resonance (^1^H-NMR).

The FT-IR is a technique easy to be employed by request little preparation to the sample and give us information about its organic chemical profile regarding the rotational-vibrational frequency from the chemical bonds present in the molecules being a useful tool in metabolomics [8].

The ^1^H-NMR has been proved for long to be also a powerful tool in metabolomics [9], [10], assessing the information related to the structure from the molecules in our sample that contain hydrogen as the large majority of organic compounds.

Such multi-omics dataset was split in two groups of data: one derived from metagenomics aggregating the OTUs from Archaea, 16S rRNA and 18S rRNA forming our organismal community matrix (matrix community) and the second group of data formed by the ionomic information acquired by ICP-OES joint to the metabolomic information represented by the integration of FT-IR and ^1^H-NMR spectra, thus being fused to one matrix of chemical profile (matrix chemicals).

As in our method we are not able to differentiate whether the cells were dead or alive in the exact time of sampling and some organisms may feed on dead cells it was assumed that all matter including the cells constituents took part of the environmental condition, therefore, regarded on the chemical profile.

The integrated statistical analysis is the tool to connect and frame the various trends acting underneath the broader complexity of the totality of the environment. Our group has being developing statistical tools to grasp the explainable features present on diverse environments [11], [12].

Here, we filter, organize and frame the data applying the pipeline of the BGC typing to expose the links amongst the organismal community and the chemical profile. It optimizes the set of information retrieved by filtering the data according to the strength of the correlation and individualizes sub-systems of organismal consortia along its chemical features framing our BGC types. Each BGC type thus comprises a small universe statistically isolated working within the environment, helping the understanding of the whole system.

The BGC typing pipeline is based on integrative statistical analysis: namely, Spearman correlation [13], [14], least-squares structuring [15] and k-means clustering [16], [17]. The set formed by the groups of organisms and the chemical profiles associated by this pipeline we call the BGC types which are meaningful a priori only in the specific study, nevertheless we expect to find similar BGC types spread on similar environments under similar conditions and analyses. The BGC typing then would improve and develop itself as more and more studies are done following this approach.

**Figure 1:**
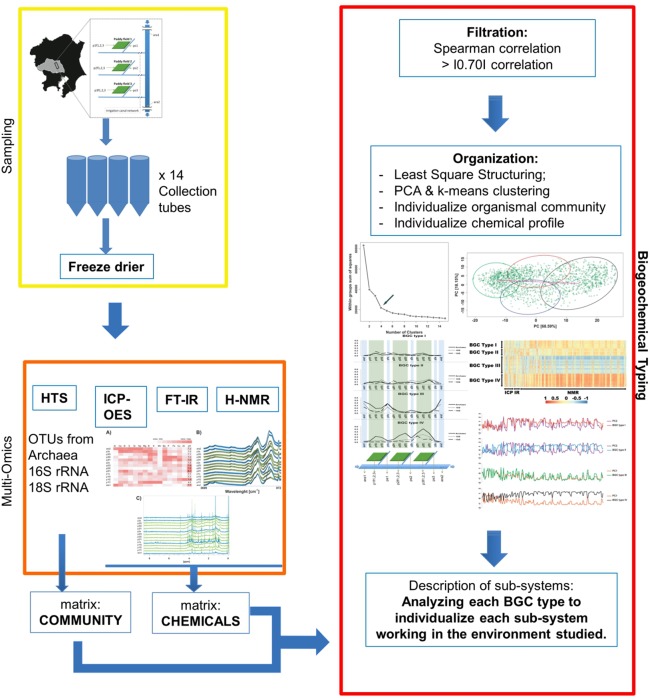
Schematic representation for the Biogeochemical Typing (BGC typing). Yellow box: steps for collection and pre-processing the samples. Orange box: steps for data acquisition and formatting for BGC typing. Red box: steps for BGC typing as the integrated statistical analyses.

The description of four singular BGC types found in this study shows that we successfully established the technique of BGC typing as a tool to characterize sub-systems composed by the community distributions and structures associated to chemical profiles on a complex environment such the Japanese paddy field and surrounding waters (Fig. 1).

## Materials and Methods

### Sampling

We designed the sampling method to encompass what we regarded as three unities of sampling sites which comprised three samples from the water of a chosen paddy field plus its collector stream. We added two sampling points from the river that boundaries the paddy fields, the Ara river – one sampling was taken from the river right before it meets the paddy field area and another right after such paddy area ends.

**Figure 2:**
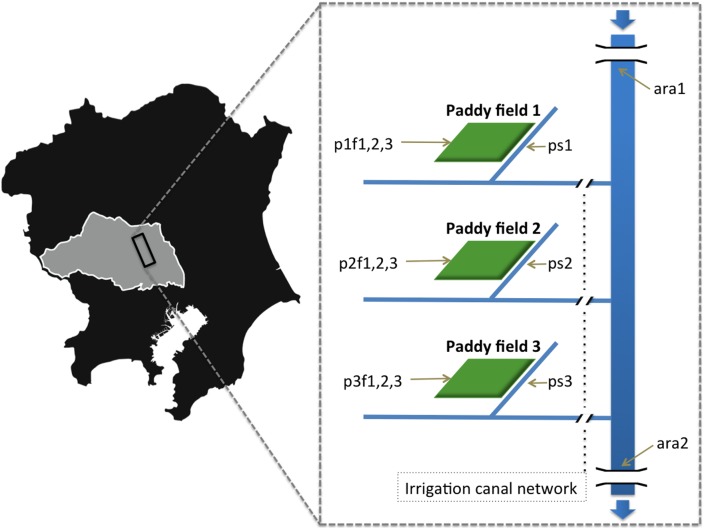
Schematic representation for the sampling location. Shadow map showing Kanto region (Japan). Grey area is Saitama prefecture. The rectangle indicates where the samples were taken. Magnified area is schematic map for the sampling site. At right side, schematic figure: Ara River was sampled in two points (ara1 and ara2) as well as paddy fields located within the area between these two river sampling points. Three independent paddy fields (paddy field 1, 2, 3) were selected and three samples were taken from each paddy field (p1f1-3, p2f1-3 and p3f1-3). These paddy fields were connected with Ara River through independent collector streams which were also sampled (p1s, p2s, p3s), respectively for each paddy field. Blue arrows indicate flow direction of Ara river and light brown arrows indicate each sampling points. Gaps in Ara River indicate bridges.

The sampling site lies on the plains of the Hiki District of Saitama Prefecture (Japan) over a large agricultural area following the course of the Ara River for approximately 13 km. Samples were collected on August 23, 2011, a few weeks prior to harvest; the paddy fields had been flooded all summer to raise the crop (rice). Using sterile tubes, four 50 mL aliquots of water were collected per sample from each sampling point. The points were located in three individual paddy fields, their respective collector streams, and the Ara River itself, for a total of 14 sampling points (Fig. 2). Specifically, there were two samples from the Ara River, one upstream of the paddy field areas and the other downstream of the paddy fields (ara1 - 36°2′32″N 139°30′8″E; ara2 - 35°56′54″N 139°32′41″E); three samples from different points of paddy field 1 (p1f1–p1f3 - 36°2′23″N 139°29′51″E) and its collector stream (p1s - 36°2′25″N 139°29′46″E); three samples from different points of paddy field 2 (p2f1–p2f3 - 35°59′37″N 139°30′5″E) and its collector stream (p2s - 35°59′37″N 139°30′5″E); three samples from different points of paddy field 3 (p3f1–p3f3 - 35°58′29″N 139°30′33″E) and its collector stream (p3s - 35°58′29″N 139°30′32″E). The samples were stored at 4°C in a cooler box and returned to the laboratory immediately, where they were stored at –80°C. Before each analysis, a 60 h freeze-drying pre-processing step was performed. All freeze-dried samples were weighed. One 50-mL aliquot was used for DNA extraction and community analysis, another aliquot for ICP-OES, another aliquot for FT-IR, and another one for ^1^H-NMR.

### HTS of the PCR products from the ribosomal RNA gene from environmental samples

High throughput sequencing is a powerful tool to identify the constituent organisms in environmental studies [18].

The DNA was extracted from the freeze-dried samples by using the Power Soil DNA extraction kit (MoBio, CA, USA); the concentration of extracted nucleic acids was measured using a CLUBIO Micro Spectrophotometer.

We amplified by PCR the small sub-unit ribosomal RNA sequences from the extracted DNA. Fragments from 16S rRNA, 18S rRNA, and Archaeal rRNA were separately amplified. The hipervariable regions from V1 to V3 for the 16S rRNA were amplified using modified Ba27F (5′-AGAGTTTGATCCTGGCTCAG-3′) as the forward primer [19] and PRUN518 (5′- ATTACCGCGGCTGCTGG-3′) as the reverse primer [20]. The hipervariable regions from V1 to V3 for the 18S rRNA were amplified using Euk1A (5′-CTGGTTGATCCTGCCAG-3′) as the forward primer and Euk516R (5′- ACGGGGGGACCAGACTTGCCCTCC-3′) as the reverse primer [21]. The hipervariable regions from V4 to V6 for the Archaeal rRNA were amplified using the 16S Archaea-specific rRNA pair of S-D-Arch-0519-a-S-15 (5′-CAGCMGCCGCGGTAA-3′) as the forward primer and S-D-Arch-1041-a-A-18 (5′-GGCCATGCACCWCCTCTC-3′) as the reverse primer [22]. The PCR products were subjected to agarose gel electrophoresis. Correctly sized fragments were retrieved from the gel and DNA was extracted using the Wizard SV Gel and PCR Clean-Up System (Promega, WI, USA). The final DNA concentration was measured using Invitrogen Quant-iT PicoGreen sDNA Reagent and Kits (Invitrogen, CA, USA). Correct dilutions were performed using Milli-Q water. The sequencing library for HTS was prepared using the GS Junior Titanium emPCR kit (Lib-L) (454 Life Sciences, CT, USA) by following the provided protocol. The library was read by a GS Junior sequencer following standard operating procedures.

The obtained reads from each GS Junior run were treated using QIIME software [23]. We followed the “454 Overview Tutorial: de novo OTU picking and diversity analyses using 454 data” (http://qiime.org/tutorials/tutorial.html) using default settings, with the following exceptions: de novo chimera detection and Trie pre-filtering in the OTU picking step [24]; uclust_ref as the clustering method [25]; SILVA 108 of the SILVA rRNA database as a sequence reference [26]. The aligned sequences were assigned against the SILVA rRNA database. OTUs represented by a single read over all sampling points were filtered out to decrease computational demand, since our correlation method used would not be able to show any trend for a sequence read only once. OTUs generated by one set of primers (e.g., Archaea) that were aligned to other domains (e.g., Eukaryota) were also filtered out to prevent overrepresentation of organisms. To compare quantified information of each OTU amongst the sampling sites, the number of reads for each OTU was divided by the total number of reads for its sampling point in order to find the relative abundance for each OTU. This step was separately performed to the three domains studied. The resulting tables of OTUs against sampling points for Archaea rRNA, 16S rRNA, and 18S rRNA were fused to one matrix (matrix community). Although the aim of this study is not building an ultimate phylogenetic tree, neither this dataset allows such task, an emulation of a phylogenetic tree was built in QIIME [23], [27] and exported to R to plot a more visually informative tree [28]. Observing the resultant tree with mixed Bacteria and Archaea domains, we opted to reassign the 16S rRNA and Archaeal OTUs according to data from the Ribosomal Database Project (RDP) [29]. The trees were built once again after the BGC typing for comparison and the 16S rRNA, 18S rRNA, and Archaeal OTUs appeared fairly distinguished.

### Inductively Coupled Plasma Optical Emission Spectrometry (ICP-OES)

For the preparation to the analysis, we suspended in Milli-Q water each freeze-dried sample to recreate the same 50 mL initial volume. Three dilutions were prepared–1∶1, 1∶10, and 1∶100-for 6-mL aliquots in 15-mL plastic tubes. For the dilutions, elemental analysis was performed using SII model SPS 5510 CCD simultaneous ICP-OES (SII NanoTechnology Inc., Chiba, Japan) equipped with an SPS-3 auto-sampler (SII NanoTechnology Inc.) and using ICP Expert software (SII NanoTechnology Inc.). We used the Multi-Element Calibration Standards 3, 4, and 5 acquired from PerkinElmer (PerkinElmer Japan Co., Ltd., Yokohama, Japan) to calibrate the machine. A concentration of 1 mg L^−1^ of each standard dilution was used for this step. We quantified 27 chemical elements: Al, B, Ba, Be, Ca, Cd, Cr, Cs, Cu, Fe, Hg, K, Li, Mg, Mn, Na, Ni, P, Pb, Rb, S, Sb, Se, Si, Sn, Sr and Zn. Three emission wavelengths of each element were chosen to satisfy both the achievement of maximum intensities and the elimination or minimization of the interference effect for discrimination of each element in the samples. The ICP-OES operating conditions were as follows: power 1.2 kW, plasma gas flow 15 L min^−1^, auxiliary gas flow 1.5 L min^−1^, nebulizer gas flow 0.75 L min^−1^, and peristaltic pump speed 15 rpm. From the obtained data, a matrix was built using the concentration in ppm for each element against the sampling points from the average result of the optimal dilution with the optimal wavelengths.

### Attenuated Total Reflectance Fourier Transformed Infrared (FT-IR) Spectroscopy

The freeze-dried samples were pressed directly on the crystal of Nicolet 6700 FT-IR spectrometer using the ATR smart iTR accessory with a high-pressure clamp (Thermo Scientific) to measure the absorbance from 4,000 to 650 cm^−1^ at a resolution of 8 cm^−1^. The peaks were annotated according to the absorbance wavelength. Distinguishable peaks consisting of regions with overlapping chemical bond signals were annotated by more than one chemical bond (i.e., as many as needed). The region of interest (ROI) was integrated for each assigned peak using an interval with no observed overlap. From 1,200 to 849 cm^−1^ the signals for the chemical bonds were permitted to overlap, such that the peaks were assigned to more than one chemical bond candidate. The region over the interval from 847 to 650 cm^−1^ was not used for annotation due to visually present but indistinguishable peaks. The spectra were integrated using Thermo OMIC software (USA). A matrix was built by assigning the sum of the integration values to a distinct part for each assigned peak as its relative amount.

### Proton Nuclear Magnetic Resonance (^1^H-NMR)

Each freeze-dried sample was dissolved individually in a proportion of 1/9 (m/v) in 100 mM 95% deuterated phosphate buffer (100 mM KH_2_PO_4_ in 99% D_2_O, pH 7.0) with 1 mM sodium 2,2-dimethyl-2-silapentane-5-sulfonate (DSS) as the internal standard. The solution was sonicated for 5 min at room temperature in a Bioruptor Diagenode (USA). Following sonication, centrifugation was performed at 8 krpm and supernatant was transferred to a 5-mm ø NMR tube.

All spectra were recorded at 298 K on a Bruker DRU-700 spectrometer (Germany) equipped with a ^1^H inverse cryogenic probe with triple-axis gradients operating at 700.15 MHz.

The ^1^H-NMR spectra were recorded at 32,768 points over 256 scans using the Watergate pulse sequence [30]. The J-resolved spectra were recorded in 32 scans per f1 increment with a total of 32 complex f1 and 16,384 complex f2 points.

The spectra were manually phased and calibrated in the Bruker Top Spin program. Integration of spectra was performed in advanced bucketing mode in Bruker AMIX 3.5 software on manually picked peaks using bucket widths equal to 0.02 ppm to find the integration value for each peak. Two broad peaks from 3.48 to 0.78 ppm were integrated by the sum of the integration for each bucket with no visible sharp peak for the region.

The peaks of the J-resolved spectra were assigned according to the Birmingham Metabolite Library [31]. The peaks for ^1^H-NMR spectra were assigned with the help of SpinAssign [32], [33], [34]. Both assigned tables were transposed to the ^1^H-NMR bucketed table (table for the integrated peaks) based on the chemical shifts. Broad peaks were assigned to proteins, whose presence was supported by positive Bradford protein assay results. 1D-STOCSY also was performed to the annotated bins correlated to the BGC types [35]. A column for the broad peak was added to the ^1^H-NMR assigned table to complete the ^1^H-NMR matrix.

## Results and Discussion

### HTS

HTS generated 100,641 sequences for the small-subunit Archaea rRNA primers, 302,974 sequences for the small-subunit 16S rRNA general primers, and 314,632 sequences for the small-subunit 18S rRNA general primers. The sequences repeated were collapsed and then assigned with similarity ≥97%, generating 5,688 OTUs for Archaea rRNA, 24,633 OTUs for 16S rRNA (no Archaeal OTU was found among universal 16S rRNA amplicons), and 9,355 OTUs for 18S rRNA. The rarefaction analysis showed a good coverage for the organismal community (Table S1, S2, S3, Fig. S1, S2, S3).

After filtering out the reads to OTUs appearing only once over the 14 sampling points, the respective totals for retrieved OTUs were 4,019, 14,942, and 6,391. Performing BGC typing as correlation filtering process, we retrieved 854 OTUs for Archaea rRNA, 1,743 OTUs for 16S rRNA and 815 OTUs for 18S rRNA. For each BGC type, we used QIIME to build an emulation of a phylogenetic tree and software R to plot and compare how the sequences were separated amongst the products retrieved from Archaea rRNA, 16S rRNA and 18S rRNA. The result displayed a fairly distinguished distribution of the OTUs (Fig. S4, S5, S6, S7).

### ICP-OES

We tested for 27 elements and detected 15 in our samples. Aluminium was detected in all samples with a lower concentration in paddy field waters than in the river. It is known that pH affects the concentration of elements; however, no clear trend between elemental concentration and pH was observed in the current study (Table S4).

### FT-IR

**Figure 3:**
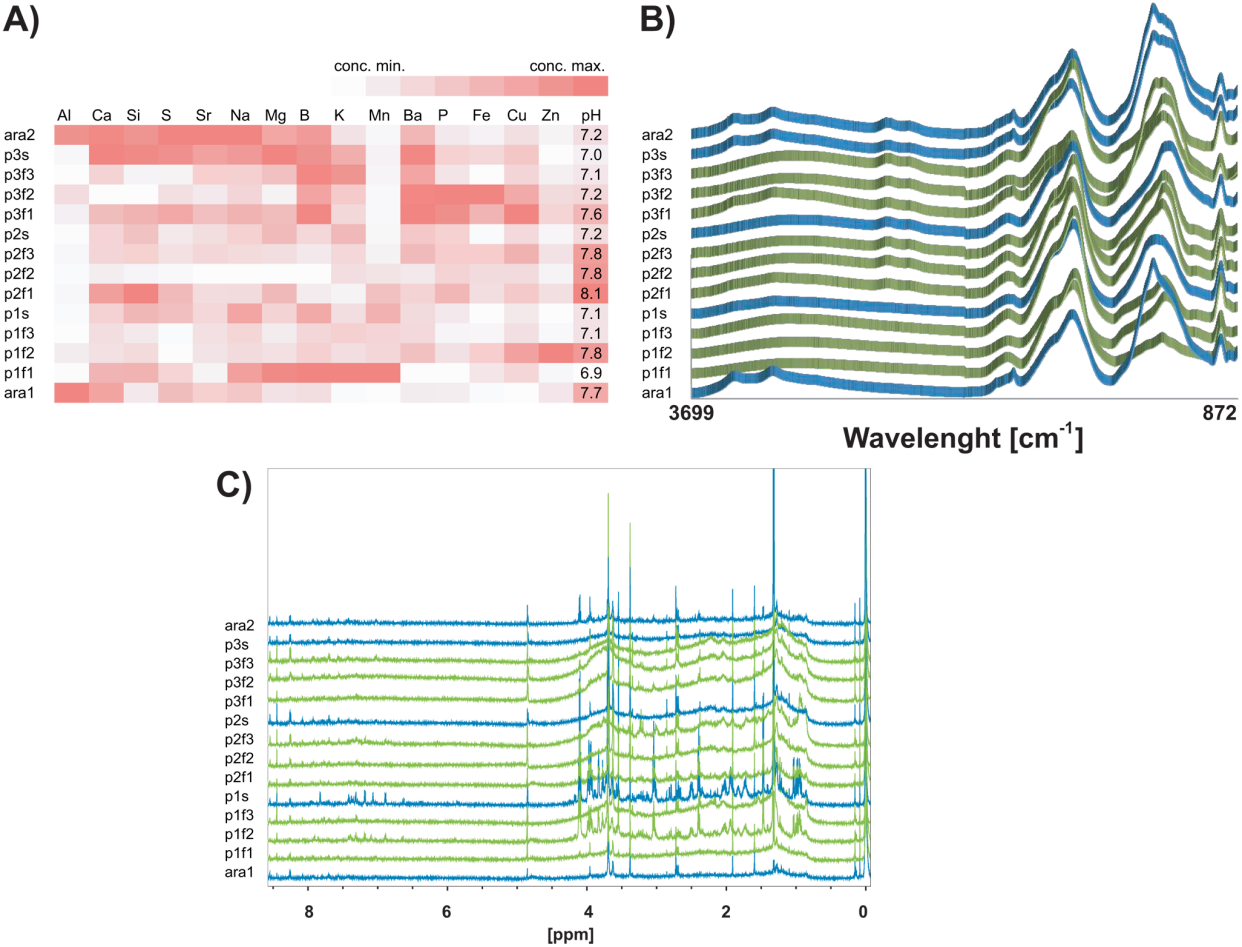
Chemical profiles for the sampling points. A) ICP-OES heatmap. X-axis: elements. Y-axis: sampling points. Red colour intensity corresponds to elemental concentration normalized by element. B) FT-IR spectra. X-axis: wavelength number. Y-axis: absorbance intensity. C) ^1^H-NMR spectra. X-axis: chemical shift. Y-axis: intensity.

We retrieved 10 distinguishable peaks and annotated accordingly [36]. The peaks annotated to NH_2_ and N-C = O follow the same pattern, suggesting that they are intrinsically linked as moieties of amino acids (Fig. 3). These peaks displayed the highest intensities at the two sampling points from the Ara River (ara1; ara2) and the one from the collector stream for paddy field 3 (p3s).

Also, we annotated on the spectra peaks as C-H and as O-H, suggesting organic energy available since molecules with aliphatic and alcohol bonds are prone to be oxidized by many organisms (Table S5).

### ^1^H-NMR

e integrated 161 individual peaks for ^1^H-NMR plus an integration for the two broad peaks, for a total of 162 ^1^H-NMR variables. The soluble organic compounds detected by this technique exhibited a clear pattern of samples from the Ara River having poorer concentrations (Fig. 3). Assignment of all peaks on the ^1^H-NMR spectra was difficult due to low concentrations of extractable compounds and to limited sensitivity insufficient to extend analysis to ^1^H-^13^C correlation experiments. However, J-resolved ^1^H-NMR analysis allowed us to annotate a total of 60 buckets (Fig. S8).

These annotations constituted molecular residue information from the chemical compounds, amino acids, or organic products present in our samples. A fraction of the peaks correlated to the BGC types was evaluated by one-dimensional statistical total correlation spectroscopy (1D-STOCSY) to verify the degree of support for the annotation [37]. The tallest peak in the spectra was assigned to lactate with good support from 1D-STOCSY plot and direct comparison against the spectrum for the pure compound provided on the Bruker AMIX database (Table S6, Fig. S9, S10).

The two broad peaks observed in the spectra had the sum of their approximated area integrated discounting the buckets with sharp peaks. These broad peaks possessed characteristic patterns of proteins [38], [39]. The Bradford assay [40] was performed on the samples with the largest broad peaks for each paddy field – p1f3, p2f3, and p3f2- with respective results of 18.1 (sd = 1.2), 29.8 (0.4), and 43.0 (0.2) µg mL^−1^ of protein. Additionally, 1D-STOCSY showed a correlation between protein concentrations against the large broad peaks in the ^1^H-NMR spectra, suggesting that soluble protein produced these peaks (Fig. S11).

### Biogeochemical typing (BGC Typing)

We constructed two independent matrices arranged by our sampling points, the matrix community and the matrix chemicals. We characterized four different groups composed by the correlated organismal and chemical profiles. We propose this statistical treatment as BGC typing.

Once the dataset is built, in this case using multi-omics approach, we applied the statistical process of BGC typing which can be divided into three main steps: 1) Filtration, 2) Organization and 3) Description.

#### 1) Filtration

The matrices formed by the OTUs retrieved from Archaea rRNA, 16S rRNA and 18S rRNA sequencing were fused in one table as being the matrix community.The matrices from ICP-OES, FT-IR, and ^1^H-NMR were fused into a single matrix termed chemicals. Using R software [41], Spearman correlation was performed between the community and chemicals matrices. The resulting correlation matrix was then imported to Microsoft Excel to extract all OTUs with coefficients equal to or higher than |0.70|.

#### 2) Organization

**Figure 4:**
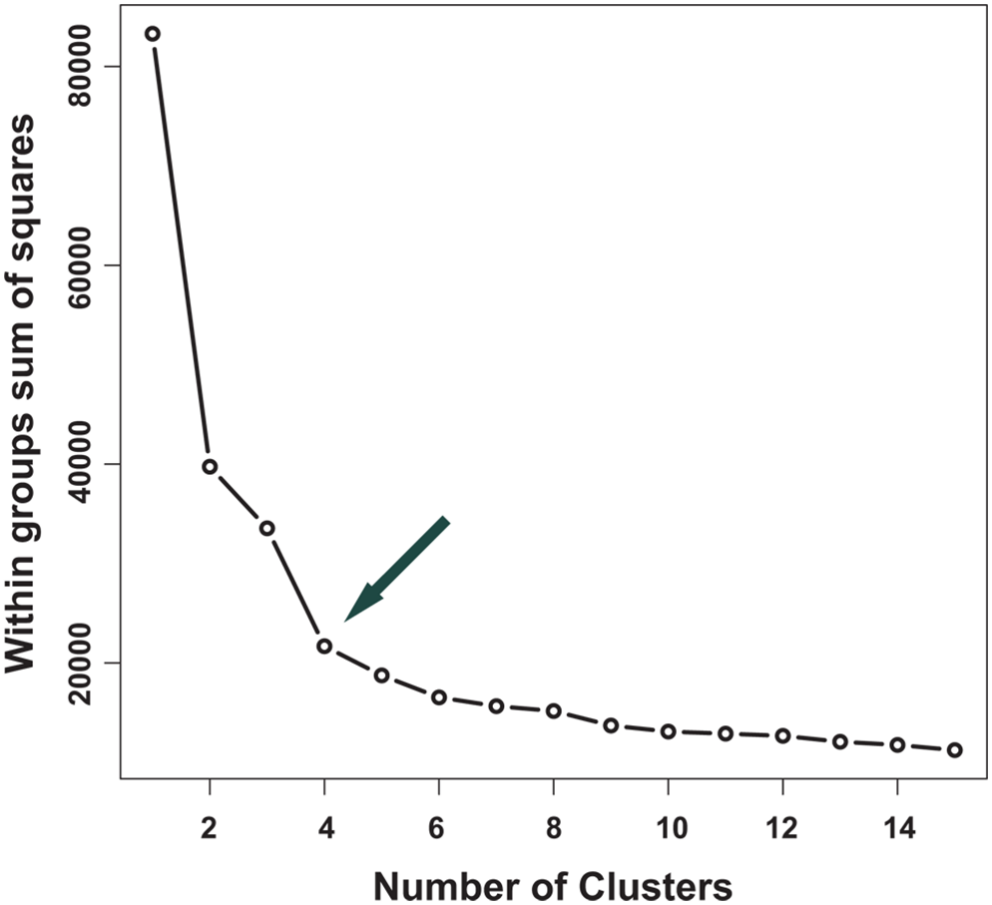
Finding the optimal number of BGC types. X-axis: number of clusters. Y-axis: sum of squared distances from each variable to the centroid within the BGC type.

To evaluate the appropriate number of BGC types representing the sub-systems, we used the least square structuring testing up to 15 groups [42], [43]. The curve inflexion indicated four as the optimal number of BGC types (Fig. 4) [15].

**Figure 5:**
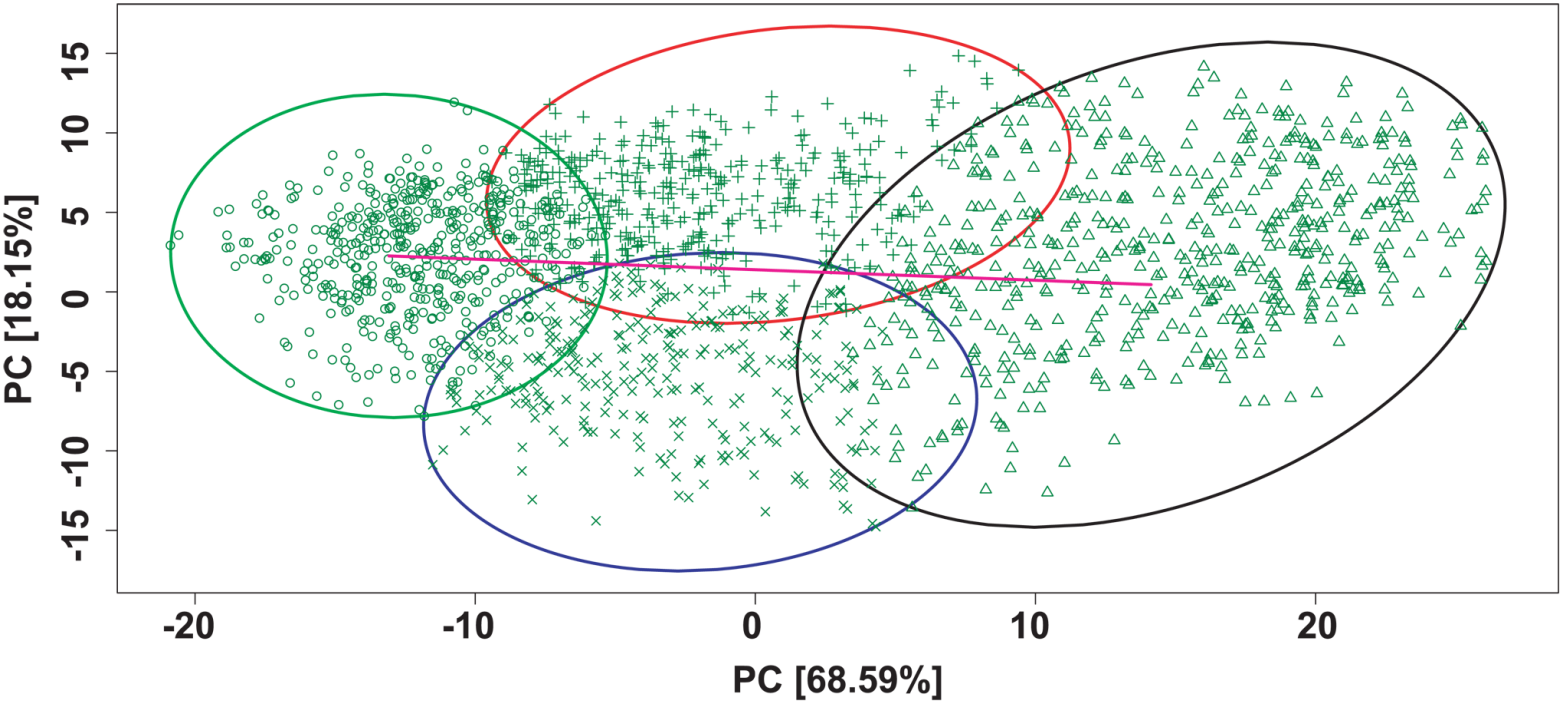
Delimiting BGC types. Plot of PCA scores for the extracted OTU matrix correlated with chemical profile. Four BGC types were delimited by k-means clustering. X-axis: PC1. Y-axis: PC2. BGC I: area enclosed in red with cross symbols, BGC II: area enclosed in blue with x symbols, BGC III: area enclosed in green with circular signals, BGC IV: area enclosed in pink with triangular signals. Arrows indicate the axes separating the BGC types; the quasi-horizontal arrow separates BGC III from BGC IV along the PC1 axis; the quasi-vertical arrow separates BGC I from BGC II along the PC2.

The correlation matrix was then divided within the principal component analysis (PCA) into four BGC types by the k-means clustering method [16] and plotted [44]. The BGC types were distributed in a cross-like fashion: two oriented horizontally according to the axis of principal component 1 (PC1) and two oriented vertically according to the axis of principal component 2 (PC2) (Fig. 5).

**Figure 6:**
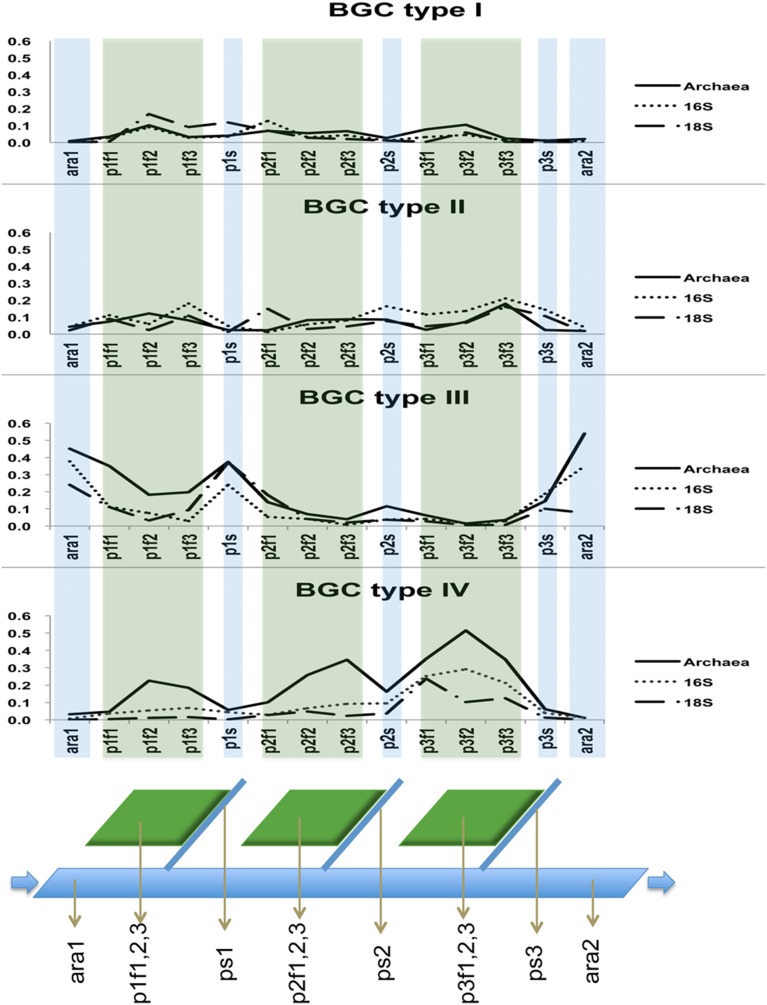
Community distributions of the BGC types over the sampling points. Distributions of relative abundances of communities identified as Archaea, 16S rRNA and 18S rRNA for the BGC types along the sampling points. X-axis: sampling points, Y-axis: relative abundance. Green shadows indicate sampling points on lentic waters and blue shadows indicate the sampling points over lotic waters. Below: a schematic drawing for the sampling points.

**Figure 7:**
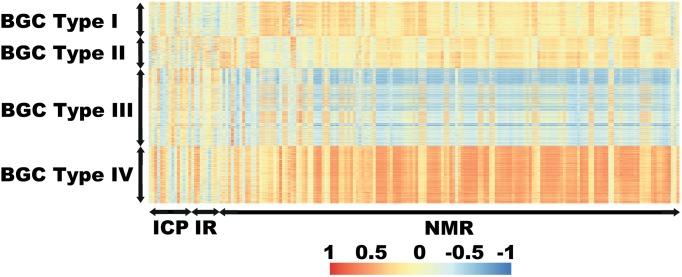
Heatmap for the Spearman correlations of extracted OTUs against chemical profiles. X-axis, in order: chemical elements (ICP-OES), wavelength number (FT-IR), chemical shifts (H-NMR). Y-axis: OTUs arranged from BGC type I (upper) to IV (bottom). BGC types are indicated on the left. The meaning of the colours is indicated in the legend at the bottom.

A table of the OTUs from each BGC type was built. For the community distribution analysis, we plotted the sum of all relative abundances along the sampling points for each BGC type (Fig. 6). To analyse the community structure, the OTUs were collapsed to the class level or beyond according to the next divergence on the taxon presented. The resulting matrices were used to find the percentage of abundance for the groups of organisms in each BGC type. To visualize the differences among chemical profiles presented by the different BGC types, we built a table and plotted (Fig. 7).

**Figure 8:**
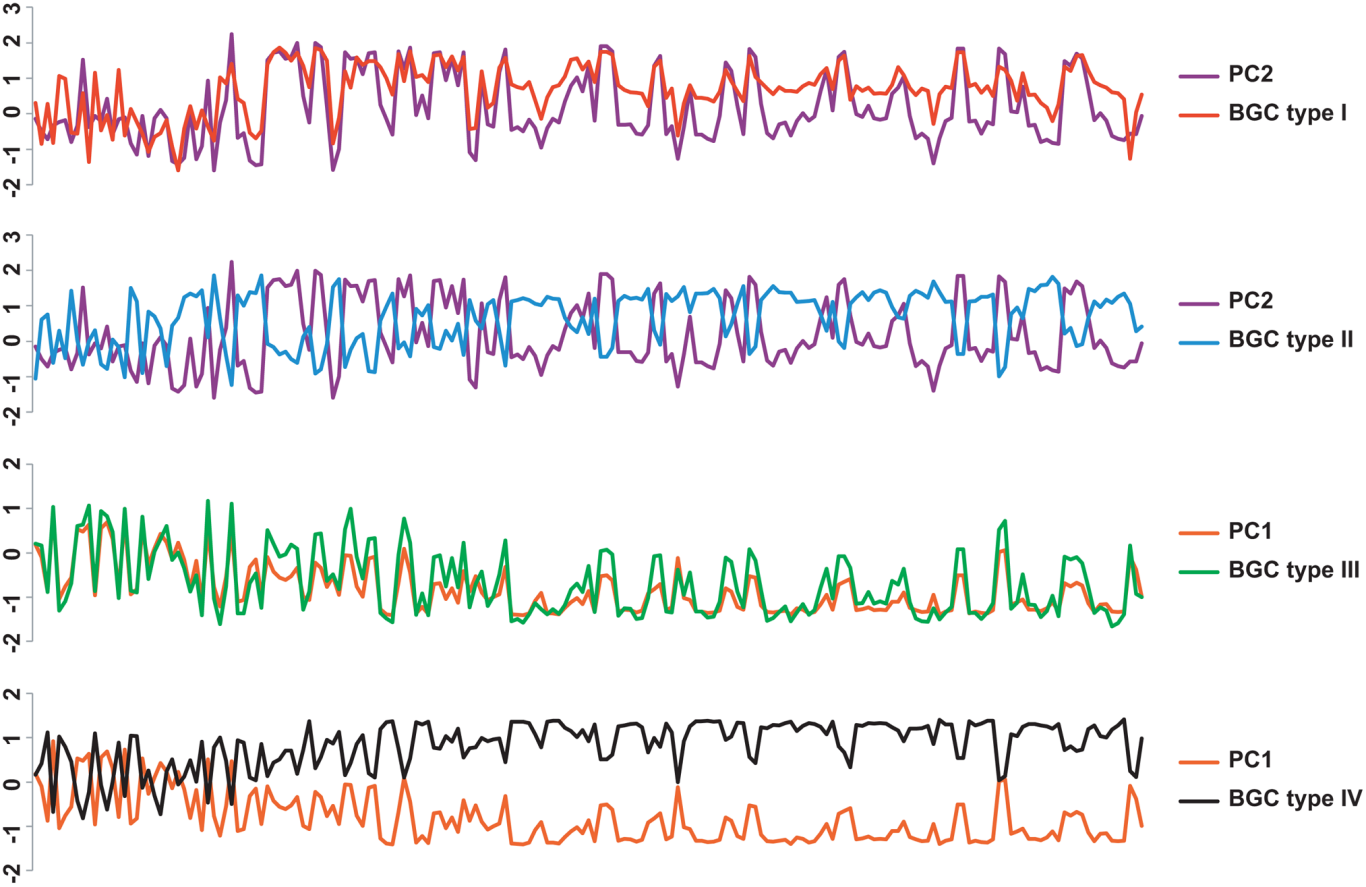
Comparison of the average correlations for each BGC type against PCA loadings. X-axis in order: chemical elements (ICP-OES), wavelength number (FT-IR), chemical shifts (H-NMR) (legend omitted). Y-axis: scaled scores. Average correlations from top to bottom are cluster 1 against PC2, cluster 2 against PC2, cluster 3 against PC1, and cluster 4 against PC1. Values are scaled by unity of variance.

A table of the chemical profile for each BGC type was built by using the average correlation index for all the chemical variables for each chemical variable from the BGC type and the loadings for PC1 or PC2 according to the one that better explains the BGC type. In order to facilitate the comparison, the tables were scaled by unity of variance without centring (Fig. 8).

The PC which better explains each BGC type had the loadings compared against the average correlations from each chemical variable using the standard deviation of a population (STDEVP). Chemical variables with STDEVP of 0.20 and over were extracted to build the table of the chemical profile for each BGC type (Table 1).

**Table 1:** Chemical profiles.

	BGC type I	BGC type II	BGC type III	BGC type IV
ICP-OES		K	Si	Ba
			Mn	Cu
			Ca	Fe
				P
				Zn
FT-IR		RC = O(1722–1647)		C-H (3037–2864)
		C = C or C-H orRS = O (879–849)		O-H (3356–3055)
^1^H-NMR	Phe (7.41, 7.39,7.37, 7.35, 7.33)	7.94	7.96	protein
	Tyr 7.19	7.70	7.84	formate 8.45
	Ser (4.00, 3.94,3.83)	7.56		Phe 7.31
	lactate (4.12,4.10, 1.32, 1.30)	7.04		Ser (4.03, 3.92, 3.80)
	Lys or Ala 3.72			pyroglutamate (4.16, 2.50, 2.47, 2.44, 2.34, 2.09, 2.07, 2.04,2.02, 2.00)
	Gly 3.55			Ala or Lys 3.69 Lys (3.75, 3.09)
	Lys (3.06, 3.04,3.01, 1.94)			Ala (3.67, 1.44)
	pyroglutamate2.41			Gly (3.58, 3.51)
	Ala (1.49, 1.46)			ketone (1.59, 1.57)
	7.84			amine or formaldehydel or methanol (3.38, 3.34)
	7.09			Val (1.10, 1.08)
	6.65			8.08 7.94 7.91 7.87 7.817.79 7.56
				7.22 7.07 7.04 7.02 4.08 4.06 3.90
				3.87 3.78 3.65 3.63 3.60 3.48 3.45
				3.42 3.40 3.32 3.28 3.26 3.22 3.20
				3.17 3.13 2.99 2.96 2.93 2.91 2.88
				2.85 2.81 2.77 2.75 2.69 2.67 2.64
				2.62 2.60 2.57 2.54 2.30 2.28 2.26
				2.22 2.20 2.16 2.12 1.89 1.86 1.84
				1.82 1.80 1.78 1.76 1.74 1.72 1.68
				1.66 1.64 1.62 1.53 1.51 1.41 1.39
				1.37 1.35 1.28 1.26 1.23 1.21 1.18
				1.16 1.13 0.92 0.89 0.87 0.84 0.82
				0.80 0.78

The chemical profile for the BGC types were divided by ICP-OES, FT-IR and ^1^H-NMR variables (omitted groups of variables are those with no high positive statistical dependence for the BGC type). Variables for ICP-OES are elements. Variables for FT-IR are the integrated area corresponding to the chemical bond in interval of wavelength (cm^−1^) showed in parentheses. Variables for ^1^H-NMR are the integrated area for the buckets (chemical shifts) in ppm; values in parentheses are chemical shifts assigned to the same compound or organic function.

#### 3) Description

According the structure presented by each BGC type regarding the organismal community and chemical profiles along the sampling points, we analysed the features that individualize them and searched for the literature that could bring sense to the sub-systems exposed.

### Organismal community distribution

Organisms of BGC type I had a scattered distribution over the sampling points, with a tendency to be less present in the lotic waters ara1, p2s, p3s and ara2 with exception of p1s. BGC type II presented a scattered distribution over the paddy field and collector streams. BGC types I and II appeared to be minimally present in the Ara River (ara1, ara2).

Organisms in BGC type III were distributed predominantly on the lotic waters and had minimum presence in the lentic waters on the paddy fields. The organisms for BGC type IV were distributed predominantly on the lentic waters from the paddy fields. Conversely, BGC type IV had a minimal distribution on the river and collector streams, the lotic waters. When we summed the three domains for each paddy field data, a gradual increase was observed from paddy 1 to paddy 3, although none of the paddies was directly connected. The organismal community distribution over the sampling points can be visualized in Fig. 6.

### Organismal community structures

The retrieved Archaeal community profile was dominated by the phylum *Euryarchaeota*, mostly composed of the classes *Methanomicrobia*, *Thermoplasmata* and *Methanobacteria*. In addition, *Euryarchaeota* exhibited the largest abundance in three out of the four BGC types, being the dominant phylum on the paddy floodwaters and the collection streams for BGC types I, II and IV. The mentioned classes tend to be directly involved in methane production [45], [46], [47]. BGC type III, which was predominantly distributed over the lotic waters of the Ara River and the collector streams, had the phylum *Crenarchaeota*, class *Thermoprotei* as the most abundant kind of Archaea. *Crenarchaeota* is suggested to take part in primary production by active involvement in the ammonia-oxidizing process of the nitrogen cycle and in autotrophic carbon assimilation [48], [49], [50]. This phylum has also been suggested to be dominant in fresh-water systems [51], which is in accordance with our findings for the distribution of BGC type III. However, such a pattern was clearly not mirrored in the paddy floodwaters, where the profile resembled that of soil [52] or a suboxic freshwater pond [53]. The BGC types distributed over the paddy fields and collector streams encompassed methanogenic candidates along with organisms that thrive in a wide range of aerobic conditions. Some of the associations might be possible by the formation of anoxic microzones in aggregates [54], although Archaea methanogens can survive in aerobic environments [55] (Fig. S12, S13, S14, S15).

The community identified by amplifying the 16S rRNA showed the ubiquitous class *Actinobacteria* to present the largest abundance (∼38%) and indeed as one of the four most abundant classes across all BGC types. It prevailed in abundance even over the ∼26% of the higher-taxon *Proteobacteria* phylum. *Actinobacteria* possess versatile metabolism, exemplified by their ability to decompose lignocellulose [56], [57] or to produce bioactive metabolites as antibiotics. The high abundance of *Actinobacteria* in BGC types II and IV in particular mirrored studies of both soil and aquatic ecosystems where this phylum often appears as an important component of the community alongside *Proteobacteria* [58], [59], [60]. Besides *Actinobacteria*, BGC types I and III shared *Proteobacteria, Flavobacteria* and *Sphingobacteria* as significant elements of the community. The bacteria detected in this study were largely from aerobic or facultative anaerobic taxa, suggesting aerobic or a micro-aerobic conditions (Fig. S16,S17, S18, S19).

The community identified amplifying the 18S rRNA, the *Eukaryota* fraction was dominated by chemoheterotrophic phyla such as *Metazoa* (with the classes *Gastrotricha* and *Rotifera* being first- and second-largest, respectively) and *Alveolata* (with the classes *Ciliophora* and *Apicomplexa* being first- and second-largest, respectively) that may serve as links in energy transfer from lower to higher trophic levels. The BGC types I, II and IV that were distributed over the paddy fields all shared this pattern. Jurgens and Gude [61] suggested that the presence of protozoans and metazoans might shape the characteristics of the bacterial community, not just regarding structure and diversity, but also triggering phenotype changes in response to predation. Similar to our findings for the Archaea community, BGC type III showed a different structure from the other BGC types, presenting the phylum *Viridiplantae* as the most abundant. *Viridiplantae* is a taxon formed by a wide range of terrestrial and aquatic primary producers that rely on chloroplasts to perform photosynthesis, which plants are part of this clade [62] (Fig. S20, S21, S22, S23).

### Chemical profiles and organismal community structures

The differences in the community distributions for BGC types I and II were not entirely clear. However, the differences in the chemical profiles revealed that all four BGC types are indeed unique. On the PCA score plot, the k-clustering method segregated four clusters as the BGC types in a cross-like fashion. This structure translates how each BGC type has its chemical profile distinguished from the others or, alternatively, its own environmental condition. In accordance with the results, BGC types III and IV were more distinct from each other than BGC types I and II were from each other, as the former were split along the PC1 axis and the latter along the PC2 axis as seen in Fig. 5. So that, BGC types III and IV had the average correlations for the chemical variables compared against PC1 while BGC I and II had the average correlations for the chemical variables compared against PC2 as seen in Fig. 8.

The chemical variables with positive correlations were extracted and assigned to the related BGC type. We listed only variables with positive correlations for each BGC type, since positivity implies that the organisms living under those specific environmental conditions are at least tolerant to those conditions. A negative correlation would not necessarily imply suppression arising from either side, biological or chemical, since the samples are from an open system with many variables that were not measured, such as geographical configuration or even weather conditions; however, it does imply the absence of a given chemical variable in the presence of a given organism, and vice-versa.

BGC type I was statistically independent from the elements tested and from the variation on the FT-IR profile. However, it presented a positive correlation to buckets on the aromatic side of the ^1^H-NMR spectra, including those annotated as phenylalanine, some annotated as other amino acids, and, most remarkably, to the largest peak on the ^1^H-NMR spectra, which was assigned as lactate. The presence of lactate suggests anoxic conditions where this molecule could be derived from pyruvate fermentation [63] as an adaptation of Metazoan organisms to thrive under suboxic conditions [64]. Despite a suggestion of anoxic conditions for this BGC type, we found that 20% of the 16S rRNA community were from chloroplasts. Therefore, we hypothesize that anoxic microzones might buffer the chloroplast-produced oxygen [65]. Another possibility is that the chloroplasts might even simply constitute debris, just dead organic material serving as a substrate to other organisms [66].

BGC type II presented the highest negative correlation to the buckets assigned to lactate on ^1^H-NMR. Since BGC types I and II inhabit the same set of samples to different degrees of colonization, this finding may suggest either competitive interactions between them or chemical heterogeneity in the occurrence of microzones displaying different chemical compositions, and hence a resulting difference in the kinds of life supported.

Organisms from BGC type III showed a positive correlation with three elements tested using ICP-OES and with two non-annotated buckets in the aromatic region of the ^1^H-NMR. One of the elements was manganese and it is a key element to, among other biological functions, photosynthesis [67], [68]. The other two elements were silica and calcium that can be linked to, but not restricted to, silification and calcification: both are processes related to increasing physical resistance in organisms as cytoskeletons and shells, features which are important in many groups within algae and microalgae, for example [69]. Indeed, for this BGC type, the phylum *Alveolata* within Eukaryota had the class *Dinophyceae* as its dominant class, instead of *Ciliophora*, which dominated the same phylum in the other BGC types. BGC type III encompassed the organisms thriving in the environments poorest in the organic compounds detected by ^1^H-NMR and FT-IR. The phyla *Viridiplantae* and *Crenarchaeota* would be the primary sources of organic carbon, with the latter also potentially serving as a nitrogen source. As the waters from paddy fields can drain into the river but the opposite is not possible, BGC type III could not act as a source of organic matter for the other BGC types. The richness in *Crenarchaeota* despite poor organic load is consistent with the findings of Ochsenreiter et al. [70], where the phylum was found throughout many kinds of environmental soil and freshwater. Despite poor organic matter content, BGC type III had the highest abundance of the three domains compared to all other BGC types, suggesting efficient cycling of photosynthesized compounds.

BGC type IV presented the group of organisms statistically correlated to more chemical variables than any of the previous ones. There are correlations to five elements within ICP-OES – barium, copper, iron, zinc and phosphorous. Sanchez-Moral et al. [71] suggested that barium precipitation can be bio-induced in pure cultures of *Actinobacteria* which was the most abundant class of bacteria present in this BGC type. Copper, iron and zinc are some of the trace elements essential for enzymatic activity in methanogenic systems, and deficiencies are suggested to diminish such activity [72]. Phosphorous is abundant in several metabolic pathways, being involved in structural biomolecules and the energy currency ATP. BGC type IV was also correlated to those bands of absorbance in FT-IR assigned to be C-H and O-H bonds: the former may suggest long carbon chains from lipids, and the latter sugar- or alcohol-related molecules. Both of them are intrinsically linked to high levels of chemical energy, either implicated in catabolism or anabolism. Within ^1^H-NMR, BGC type IV was correlated to a vast part of the spectra, suggesting a chemically rich organic environment, with relationships to many buckets assigned to amino acids and to the broad peaks assigned as protein. This would suggest nitrogen recycling, meaning the group presents an organismal community that may be both source and consumer of the organic compound assigned as protein.

## Conclusions

After the evaluation done for each BGC type, we can classify them roughly to the remarkable features that individualize them. According to the findings, BGC type I would represent the sub-system represented by the anoxic/near anoxic microzones by presenting lactate as an important energy source intermediate (aka the “BGC type Anoxic”). The BGC type II would be the aerophilic counter part of BGC type I, eventually presenting a relation energy transfer between them (aka the “BGC type Counter Part”). The BGC type III would represent a sub-system of photosynthetic organisms and the related chemical profile (aka the “BGC type Photosynthetic”). The BGC type IV would represent the most active in the cycling of organic compounds (aka the “BGC type Glutton”).

Hence, we successfully established the BGC typing analysis pipeline technique and applied to the environment of Japanese paddy fields bringing forth four unique subset of organismal and chemical assembles. The technique is flexible and can accept any biogeochemical or omics measurements, since it operates on numerical tables of values (e.g., intensity, concentration, number of reads, etc.) and can contribute to further insights into biogeochemical cycles in other environments.

This holistic technique will broaden the understanding of “hidden” sub-systems working within the totality of the environments.

### Ethics Statement

There is no specific permission required for all of following sampling points as they are public places. Also the field does not host endangered or protected species. The exact location for the sampling points from Ara River (ara1, ara2) are 36°2′32″N 139°30′8″E and 35°56″54″N 139°32′41″E respectively, from paddy field 1 (p1f1–p1f3) are: 36°2′23″N 139°29′51″E, from paddy field 2 (p2f1–p2f3) are: 35°59′37″N 139°30′5″E, from paddy field 3 (p3f1–p3f3) are: 35°58′29″N 139°30′33″E, from collector stream from paddy field 1 (p1s) is: 36°2′25″N 139°29′46″E, from collector stream from paddy field 2 (p2s) is: 35°59′37″N 139°30′5″E and from collector stream from paddy field 3 (p3s) is: 35°58′29″N 139°30′32″E.

The whole of the sequences retrieved in our study are available in the DDBJ Sequenced Read Archive under accession number DRA002437.

## Supporting Information

Figure S1 **Rarefaction curve to total Archaea OTUs.** Rarefaction curve for Archaea rRNA OTUs for all samples. X-axis: number of readings. Y-axis: number of species (log).
(PDF)

Figure S2 **Rarefaction curve to toatal 16S rRNA OTUs.** Rarefaction curve for 16S rRNA OTUs for all samples. X-axis: number of readings. Y-axis: number of species (log).
(PDF)

Figure S3 **Rarefaction curve to total 18S rRNA OTUs.** Rarefaction curve for 18S rRNA OTUs for all samples. X-axis: number of readings. Y-axis: number of species (log).
(PDF)

Figure S4 **Emulation of a phylogenetic tree for BGC type I.** Abundance range indicated by the size of the symbol indicated at upper right. Symbols indicative of the domain indicated at middle right. Colour codes for the most abundant classes indicated at lower right. The number of symbols for each branch is related to the number of sampling points within the OTUs present.
(PDF)

Figure S5 **Emulation of a phylogenetic tree for BGC type II.** Abundance range indicated by the size of the symbol indicated at upper right. Symbols indicative of the domain indicated at middle right. Colour codes for the most abundant classes indicated at lower right. The number of symbols for each branch is related to the number of sampling points within the OTUs present.
(PDF)

Figure S6 **Emulation of a phylogenetic tree for BGC type III.** Abundance range indicated by the size of the symbol indicated at upper right. Symbols indicative of the domain indicated at middle right. Colour codes for the most abundant classes indicated at lower right. The number of symbols for each branch is related to the number of sampling points within the OTUs present.
(PDF)

Figure S7 **Emulation of a phylogenetic tree for BGC type IV.** Abundance range indicated by the size of the symbol indicated at upper right. Symbols indicative of the domain indicated at middle right. Colour codes for the most abundant classes indicated at lower right. The number of symbols for each branch is related to the number of sampling points within the OTUs present.
(PDF)

Figure S8 **J-resolved ^1^H-NMR with annotations.** J-resolved ^1^H-NMR analysis for aromatic and non-aromatic regions of the spectra with annotations.
(PDF)

Figure S9 **1D-STOCSY correlation for chemical shift 1.32 ppm.** 1D-STOCSY with centroid at 1.32 ppm showing high correlation with the other chemical shifts from the lactate assignment. X-axis: chemical shifts. Y-axis: degree of correlation. Colours simplify visualization: cold colours indicate negative correlations and hot colours indicate positive correlations.
(TIF)

Figure S10 Comparison between the ^1^H-NMR spectra against the Bruker AMIX database for the pure compound lactate (lactic acid). The ^1^HNMR spectra with lower baselines are from this study. The upward-shifted baseline spectrum in orange is the lactate spectrum provided in Bruker AMIX software. Numbers indicate chemical shift.
(PDF)

Figure S11 **1D-STOCSY correlation for the integration annotated as protein.** 1D**-**STOCSY with centroid at the region of interest integrated and annotated as protein showing high correlation with most of the chemical shifts from the spectra. X-axis: chemical shifts. Y-axis: degree of correlation. Colours simplify visualization: cold colours indicate negative correlations and hot colours indicate positive correlations.
(TIF)

Figure S12 **Percentage of Archaea OTUs for BGC type I.** Archaeal OTUs for BGC type I collapsed to the class level or beyond according to the next divergence on the taxon presented. The four most abundant taxa are shown, with others collapsed.
(PDF)

Figure S13 **Percentage of Archaea OTUs for BGC type II.** Archaeal OTUs for BGC type II collapsed to the class level or beyond according to the next divergence on the taxon presented. The four most abundant taxa are shown, with others collapsed.
(PDF)

Figure S14 **Percentage of Archaea OTUs for BGC type III.** Archaeal OTUs for BGC type III collapsed to the class level or beyond according to the next divergence on the taxon presented. The four most abundant taxa are shown, with others collapsed.
(PDF)

Figure S15 **Percentage of Archaea OTUs for BGC type IV.** Archaeal OTUs for BGC type IV collapsed to the class level or beyond according to the next divergence on the taxon presented. The four most abundant taxa are shown, with others collapsed.
(PDF)

Figure S16 **Percentage of 16S rRNA OTUs for BGC type I.** 16S OTUs for BGC type I collapsed to the class level or beyond according to the next divergence on the taxon presented. The four most abundant taxa are shown, with others collapsed.
(PDF)

Figure S17 **Percentage of 16S rRNA OTUs for BGC type II.** 16S OTUs for BGC type II collapsed to the class level or beyond according to the next divergence on the taxon presented. The four most abundant taxa are shown, with others collapsed.
(PDF)

Figure S18 **Percentage of 16S rRNA OTUs for BGC type III.** 16S rRNA OTUs for BGC type III collapsed to the class level or beyond according to the next divergence on the taxon presented. The four most abundant taxa are shown, with others collapsed.
(PDF)

Figure S19 **Percentage of 16S rRNA OTUs for BGC type IV.** 16S rRNA OTUs for BGC type IV collapsed to the class level or beyond according to the next divergence on the taxon presented. The four most abundant taxa are shown, with others collapsed.
(PDF)

Figure S20 **Percentage of 18S rRNA OTUs for BGC type I.** 18S OTUs for BGC type I collapsed to the class level or beyond according to the next divergence on the taxon presented. The four most abundant taxa are shown, with others collapsed.
(PDF)

Figure S21 **Percentage of 18S rRNA OTUs for BGC type II.** 18S OTUs for BGC type II collapsed to the class level or beyond according to the next divergence on the taxon presented. The four most abundant taxa are shown, with others collapsed.
(PDF)

Figure S22 **Percentage of 18S rRNA OTUs for BGC type III.** 18S rRNA OTUs for BGC type III collapsed to the class level or beyond according to the next divergence on the taxon presented. The four most abundant taxa are shown, with others collapsed.
(PDF)

Figure S23 **Percentage of 18S rRNA OTUs for BGC type IV.** 18S rRNA OTUs for BGC type IV collapsed to the class level or beyond according to the next divergence on the taxon presented. The four most abundant taxa are shown, with others collapsed.
(PDF)

Table S1 **Archaea OTUs in number of reads along sampling points.** Rows: OTUs. Columns: sampling points.
(XLSX)

Table S2 **16S OTUs in number of reads along sampling points.** Rows: OTUs. Columns: sampling points.
(XLSX)

Table S3 **18S OTUs in number of reads along sampling points.** Rows: OTUs. Columns: sampling points.
(XLSX)

Table S4 **Elemental concentrations along sampling points.** Rows: sampling points. Columns: elements and pH.
(XLSX)

Table S5 **Infrared absorbance along sampling points.** Rows: sampling points. Columns: annotated regions of interest in intervals of wavelength (cm^−1^) indicated in parentheses.
(XLSX)

Table S6 **^1^H-NMR along sampling points.** Rows: sampling points. Columns: bins for chemical shifts (ppm) with annotation where applicable and integration for the broad peaks annotated as protein.
(XLS)

## References

[1] Asakura T, Date Y, Kikuchi J (2014) Comparative analysis of chemical and microbial profiles in estuarine sediments sampled from Kanto and Tohoku regions in Japan. Anal Chem 86: 5425–5432. 24889864 10.1021/ac5005037

[2] Joyce AR, Palsson B (2006) The model organism as a system: integrating 'omics' data sets. Nat Rev Mol Cell Biol 7: 198–210. 16496022 10.1038/nrm1857

[3] Enjalbert B, Jourdan F, Portais JC (2011) Intuitive Visualization and Analysis of Multi-Omics Data and Application to Escherichia coli Carbon Metabolism. Plos One 6. 10.1371/journal.pone.0021318 PMC3120879 21731702

[4] Castell WZ, Ernst D (2012) Experimental 'omics' data in tree research: facing complexity. Trees-Structure and Function 26: 1723–1735.

[5] Xie J, Hu L, Tang J, Wu X, Li N, et al (2011) Ecological mechanisms underlying the sustainability of the agricultural heritage rice-fish coculture system. Proceedings of the National Academy of Sciences of the United States of America 108: E1381–E1387. 22084110 10.1073/pnas.1111043108 PMC3250190

[6] Caporaso JG, Lauber CL, Walters WA, Berg-Lyons D, Lozupone CA, et al (2011) Global patterns of 16S rRNA diversity at a depth of millions of sequences per sample. Proc Natl Acad Sci U S A 108 Suppl 1 4516–4522. 20534432 10.1073/pnas.1000080107 PMC3063599

[7] Hirschi KD (2003) Strike while the ionome is hot: making the most of plant genomic advances. Trends Biotechnol 21: 520–521. 14624858 10.1016/j.tibtech.2003.09.013

[8] Sitole L, Steffens F, Krüger TP, Meyer D (2014) Mid-ATR-FTIR Spectroscopic Profiling of HIV/AIDS Sera for Novel Systems Diagnostics in Global Health. OMICS 18: 513–523. 24937213 10.1089/omi.2013.0157 PMC4108936

[9] Kikuchi J, Shinozaki K, Hirayama T (2004) Stable isotope labeling of Arabidopsis thaliana for an NMR-based metabolomics approach. Plant Cell Physiol 45: 1099–1104. 15356336 10.1093/pcp/pch117

[10] Everroad RC, Yoshida S, Tsuboi Y, Date Y, Kikuchi J, et al. (2012) Concentration of metabolites from low-density planktonic communities for environmental metabolomics using nuclear magnetic resonance spectroscopy. J Vis Exp: e3163. 10.3791/3163 PMC3580268 22508363

[11] Ogata Y, Chikayama E, Morioka Y, Everroad RC, Shino A, et al (2012) ECOMICS: a web-based toolkit for investigating the biomolecular web in ecosystems using a trans-omics approach. PLoS One 7: e30263. 22319563 10.1371/journal.pone.0030263 PMC3271069

[12] Nakanishi Y, Fukuda S, Chikayama E, Kimura Y, Ohno H, et al (2011) Dynamic omics approach identifies nutrition-mediated microbial interactions. J Proteome Res 10: 824–836. 21058740 10.1021/pr100989c

[13] Gottel NR, Castro HF, Kerley M, Yang Z, Pelletier DA, et al (2011) Distinct microbial communities within the endosphere and rhizosphere of Populus deltoides roots across contrasting soil types. Appl Environ Microbiol 77: 5934–5944. 21764952 10.1128/AEM.05255-11 PMC3165402

[14] Wubet T, Christ S, Schöning I, Boch S, Gawlich M, et al (2012) Differences in soil fungal communities between European beech (Fagus sylvatica L.) dominated forests are related to soil and understory vegetation. PLoS One 7: e47500. 23094057 10.1371/journal.pone.0047500 PMC3475711

[15] Mirkin B (1998) Least-Squares Structuring, Clustering and Data Processing Issues. The Computer Journal 41: 518–536.

[16] Maechler M (2013) cluster: Cluster Analysis Basics and Extensions. R package version 1.14.4. In: Rousseeuw P, Hubert M, Hornik K, editors.

[17] Dardenne F, Van Dongen S, Nobels I, Smolders R, De Coen W, et al (2008) Mode of action clustering of chemicals and environmental samples on the bases of bacterial stress gene inductions. Toxicological Sciences 101: 206–214. 17951611 10.1093/toxsci/kfm262

[18] Margulies M, Egholm M, Altman WE, Attiya S, Bader JS, et al (2005) Genome sequencing in microfabricated high-density picolitre reactors. Nature 437: 376–380. 16056220 10.1038/nature03959 PMC1464427

[19] Weisburg WG, Barns SM, Pelletier DA, Lane DJ (1991) 16S Ribosomal DNA Amplification for Phylogenetic Study. Journal of Bacteriology 173: 697–703. 1987160 10.1128/jb.173.2.697-703.1991 PMC207061

[20] Muyzer G, Dewaal EC, Uitterlinden AG (1993) Profiling of Complex Microbial-Populations by Denaturing Gradient Gel-Electrophoresis Analysis of Polymerase Chain Reaction-Amplified Genes-Coding for 16s Ribosomal-RNA. Applied and Environmental Microbiology 59: 695–700. 7683183 10.1128/aem.59.3.695-700.1993 PMC202176

[21] Diez B, Pedros-Alio C, Marsh TL, Massana R (2001) Application of denaturing gradient gel electrophoresis (DGGE) to study the diversity of marine picoeukaryotic assemblages and comparison of DGGE with other molecular techniques. Applied and Environmental Microbiology 67: 2942–2951. 11425706 10.1128/AEM.67.7.2942-2951.2001 PMC92965

[22] Klindworth A, Pruesse E, Schweer T, Peplies J, Quast C, et al. (2013) Evaluation of general 16S ribosomal RNA gene PCR primers for classical and next-generation sequencing-based diversity studies. Nucleic Acids Research 41. 10.1093/nar/gks808 PMC3592464 22933715

[23] Caporaso JG, Kuczynski J, Stombaugh J, Bittinger K, Bushman FD, et al (2010) QIIME allows analysis of high-throughput community sequencing data. Nature Methods 7: 335–336. 20383131 10.1038/nmeth.f.303 PMC3156573

[24] Bonder MJ, Abeln S, Zaura E, Brandt BW (2012) Comparing clustering and pre-processing in taxonomy analysis. Bioinformatics 28: 2891–2897. 22962346 10.1093/bioinformatics/bts552

[25] Edgar RC (2010) Search and clustering orders of magnitude faster than BLAST. Bioinformatics 26: 2460–2461. 20709691 10.1093/bioinformatics/btq461

[26] Quast C, Pruesse E, Yilmaz P, Gerken J, Schweer T, et al (2013) The SILVA ribosomal RNA gene database project: improved data processing and web-based tools. Nucleic Acids Research 41: D590–D596. 23193283 10.1093/nar/gks1219 PMC3531112

[27] Izquierdo-Carrasco F, Smith SA, Stamatakis A (2011) Algorithms, data structures, and numerics for likelihood-based phylogenetic inference of huge trees. Bmc Bioinformatics 12. 10.1186/1471-2105-12-470 PMC3267785 22165866

[28] McMurdie PJ, Holmes S (2013) phyloseq: An R Package for Reproducible Interactive Analysis and Graphics of Microbiome Census Data. Plos One 8. 10.1371/journal.pone.0061217 PMC3632530 23630581

[29] Wang Q, Garrity GM, Tiedje JM, Cole JR (2007) Naive Bayesian classifier for rapid assignment of rRNA sequences into the new bacterial taxonomy. Applied and Environmental Microbiology 73: 5261–5267. 17586664 10.1128/AEM.00062-07 PMC1950982

[30] Date Y, Iikura T, Yamazawa A, Moriya S, Kikuchi J (2012) Metabolic sequences of anaerobic fermentation on glucose-based feeding substrates based on correlation analyses of microbial and metabolite profiling. J Proteome Res 11: 5602–5610. 23110341 10.1021/pr3008682

[31] Ludwig C, Easton JM, Lodi A, Tiziani S, Manzoor SE, et al (2012) Birmingham Metabolite Library: a publicly accessible database of 1-D H-1 and 2-D H-1 J-resolved NMR spectra of authentic metabolite standards (BML-NMR). Metabolomics 8: 8–18.

[32] Akiyama K, Chikayama E, Yuasa H, Shimada Y, Tohge T, et al (2008) PRIMe: a Web site that assembles tools for metabolomics and transcriptomics. In Silico Biol 8: 339–345. 19032166

[33] Chikayama E, Sekiyama Y, Okamoto M, Nakanishi Y, Tsuboi Y, et al (2010) Statistical indices for simultaneous large-scale metabolite detections for a single NMR spectrum. Anal Chem 82: 1653–1658. 20128615 10.1021/ac9022023

[34] Chikayama E, Suto M, Nishihara T, Shinozaki K, Kikuchi J (2008) Systematic NMR analysis of stable isotope labeled metabolite mixtures in plant and animal systems: coarse grained views of metabolic pathways. PLoS One 3: e3805. 19030231 10.1371/journal.pone.0003805 PMC2583929

[35] Edoardo G (2012) muma: Metabolomics Univariate and Multivariate Analysis. R package version 1.4. In: Francesca C, Silvia M, Andrea S, Michela G, editors.

[36] Ogura T, Date Y, Kikuchi J (2013) Differences in cellulosic supramolecular structure of compositionally similar rice straw affect biomass metabolism by paddy soil microbiota. PLoS ONE 8: e66919. 23840554 10.1371/journal.pone.0066919 PMC3686774

[37] Cloarec O, Dumas ME, Craig A, Barton RH, Trygg J, et al (2005) Statistical total correlation spectroscopy: An exploratory approach for latent biomarker identification from metabolic H-1 NMR data sets. Analytical Chemistry 77: 1282–1289. 15732908 10.1021/ac048630x

[38] Kikuchi J, Asakura T, Loach PA, Parkes-Loach PS, Shimada K, et al (1999) A light-harvesting antenna protein retains its folded conformation in the absence of protein-lipid and protein-pigment interactions. Biopolymers 49: 361–372. 10101971 10.1002/(SICI)1097-0282(19990415)49:5<361::AID-BIP3>3.0.CO;2-4

[39] Kraft BJ, Masuda S, Kikuchi J, Dragnea V, Tollin G, et al (2003) Spectroscopic and mutational analysis of the blue-light photoreceptor AppA: A novel photocycle involving flavin stacking with an aromatic amino acid. Biochemistry 42: 6726–6734. 12779327 10.1021/bi030055o

[40] Bradford MM (1976) Rapid and Sensitive Method for Quantitation of Microgram Quantities of Protein Utilizing Principle of Protein-Dye Binding. Analytical Biochemistry 72: 248–254. 942051 10.1016/0003-2697(76)90527-3

[41] Team RDC (2008) R: A Language and environment for statistical computing.: R Foundation for Statistical Computing.

[42] Peeples MA (2011) R Script for K-Means Cluster Analysis.

[43] Pollard D (1982) a Central Limit-Theorem for K-Means Clustering. Annals of Probability 10: 919–926.

[44] Kolde R (2012) pheatmap: Pretty Heatmaps. R package version 0.7.4.

[45] Sakai S, Imachi H, Hanada S, Ohashi A, Harada H, et al (2008) Methanocella paludicola gen. nov., sp nov., a methane-producing archaeon, the first isolate of the lineage 'Rice Cluster I', and proposal of the new archaeal order Methanocellales ord. nov. International Journal of Systematic and Evolutionary Microbiology 58: 929–936. 18398197 10.1099/ijs.0.65571-0

[46] Poulsen M, Schwab C, Jensen BB, Engberg RM, Spang A, et al. (2013) Methylotrophic methanogenic Thermoplasmata implicated in reduced methane emissions from bovine rumen. Nature Communications 4. 10.1038/ncomms2432 23385573

[47] Whitford MF, Teather RM, Forster RJ (2001) Phylogenetic analysis of methanogens from the bovine rumen. BMC Microbiology. 10.1186/1471-2180-1-5 PMC32158 11384509

[48] Herrmann M, Saunders AM, Schramm A (2008) Archaea dominate the ammonia-oxidizing community in the rhizosphere of the freshwater macrophyte Littorella uniflora. Applied and Environmental Microbiology 74: 3279–3283. 18344332 10.1128/AEM.02802-07 PMC2394948

[49] Wang S, Wang Y, Feng X, Zhai L, Zhu G (2011) Quantitative analyses of ammonia-oxidizing Archaea and bacteria in the sediments of four nitrogen-rich wetlands in China. Applied Microbiology and Biotechnology 90: 779–787. 21253721 10.1007/s00253-011-3090-0

[50] Pratscher J, Dumont MG, Conrad R (2011) Ammonia oxidation coupled to CO2 fixation by archaea and bacteria in an agricultural soil. Proceedings of the National Academy of Sciences of the United States of America 108: 4170–4175. 21368116 10.1073/pnas.1010981108 PMC3053987

[51] Ghai R, Rodriguez-Valera F, McMahon KD, Toyama D, Rinke R, et al. (2011) Metagenomics of the Water Column in the Pristine Upper Course of the Amazon River. Plos One 6. 10.1371/journal.pone.0023785 PMC3158796 21915244

[52] Kudo Y, Nakajima T, Miyaki T, Oyaizu H (1997) Methanogen flora of paddy soils in Japan. Fems Microbiology Ecology 22: 39–48.

[53] Briee C, Moreira D, Lopez-Garcia P (2007) Archaeal and bacterial community composition of sediment and plankton from a suboxic freshwater pond. Research in Microbiology 158: 213–227. 17346937 10.1016/j.resmic.2006.12.012

[54] Ploug H, Kuhl M, BuchholzCleven B, Jorgensen BB (1997) Anoxic aggregates - an ephemeral phenomenon in the pelagic environment? Aquatic Microbial Ecology 13: 285–294.

[55] Grossart H-P, Frindte K, Dziallas C, Eckert W, Tang KW (2011) Microbial methane production in oxygenated water column of an oligotrophic lake. Proceedings of the National Academy of Sciences of the United States of America 108: 19657–19661. 22089233 10.1073/pnas.1110716108 PMC3241779

[56] Crawford DL (1978) Lignocellulose Decomposition by Selected Streptomyces Strains. Applied and Environmental Microbiology 35: 1041–1045. 677871 10.1128/aem.35.6.1041-1045.1978 PMC242982

[57] Ball AS, Godden B, Helvenstein P, Penninckx MJ, McCarthy AJ (1990) Lignocarbohydrate Solubilization From Straw by Actinomycetes. Applied and Environmental Microbiology 56: 3017–3022. 16348309 10.1128/aem.56.10.3017-3022.1990 PMC184892

[58] Peiffer JA, Spor A, Koren O, Jin Z, Tringe SG, et al (2013) Diversity and heritability of the maize rhizosphere microbiome under field conditions. Proceedings of the National Academy of Sciences of the United States of America 110: 6548–6553. 23576752 10.1073/pnas.1302837110 PMC3631645

[59] Andreote FD, Javier Jimenez D, Chaves D, Franco Dias AC, Luvizotto DM, et al. (2012) The Microbiome of Brazilian Mangrove Sediments as Revealed by Metagenomics. Plos One 7. 10.1371/journal.pone.0038600 PMC3380894 22737213

[60] Glockner FO, Zaichikov E, Belkova N, Denissova L, Pernthaler J, et al. (2000) Comparative 16S rRNA analysis of lake bacterioplankton reveals globally distributed phylogenetic clusters including an abundant group of actinobacteria. Applied and Environmental Microbiology 66: 5053–+. 10.1128/aem.66.11.5053-5065.2000 PMC92419 11055963

[61] Jurgens K, Gude H (1994) the Potential Importance of Grazing-Resistant Bacteria in Planktonic Systems. Marine Ecology Progress Series 112: 169–188.

[62] Wodniok S, Brinkmann H, Gloeckner G, Heidel AJ, Philippe H, et al. (2011) Origin of land plants: Do conjugating green algae hold the key? Bmc Evolutionary Biology 11. 10.1186/1471-2148-11-104 PMC3088898 21501468

[63] Semenza GL (2012) Hypoxia-Inducible Factors in Physiology and Medicine. Cell 148: 399–408. 22304911 10.1016/j.cell.2012.01.021 PMC3437543

[64] Braeckman U, Vanaverbeke J, Vincx M, van Oevelen D, Soetaert K (2013) Meiofauna Metabolism in Suboxic Sediments: Currently Overestimated. Plos One 8. 10.1371/journal.pone.0059289 PMC3610736 23555652

[65] Paerl HW, Bebout BM (1988) Direct Measurement of O-2-Depleted Microzones in Marine Oscillatoria - Relation to N-2 Fixation. Science 241: 442–445. 17792609 10.1126/science.241.4864.442

[66] Kiorboe T, Tang K, Grossart HP, Ploug H (2003) Dynamics of microbial communities on marine snow aggregates: Colonization, growth, detachment, and grazing mortality of attached bacteria. Applied and Environmental Microbiology 69: 3036–3047. 12788697 10.1128/AEM.69.6.3036-3047.2003 PMC161531

[67] Kanehisa M, Goto S, Sato Y, Furumichi M, Tanabe M (2012) KEGG for integration and interpretation of large-scale molecular data sets. Nucleic Acids Research 40: D109–D114. 22080510 10.1093/nar/gkr988 PMC3245020

[68] Kanehisa M, Goto S (2000) KEGG: Kyoto Encyclopedia of Genes and Genomes. Nucleic Acids Research 28: 27–30. 10592173 10.1093/nar/28.1.27 PMC102409

[69] Brownlee C, Taylor AR (2002) Algal Calcification and Silification. Encyclopedia of Life Sciences: Macmillan Publishers Ltd, Nature Publishing Group.

[70] Ochsenreiter T, Selezi D, Quaiser A, Bonch-Osmolovskaya L, Schleper C (2003) Diversity and abundance of Crenarchaeota in terrestrial habitats studied by 16S RNA surveys and real time PCR. Environmental Microbiology 5: 787–797. 12919414 10.1046/j.1462-2920.2003.00476.x

[71] Sanchez-Moral S, Luque L, Canaveras JC, Laiz L, Jurado V, et al (2004) Bioinduced barium precipitation in St. Callixtus and domitilla catacombs. Annals of Microbiology 54: 1–12.

[72] Unal B, Perry VR, Sheth M, Gomez-Alvarez V, Chin K-J, et al. (2012) Trace elements affect methanogenic activity and diversity in enrichments from subsurface coal bed produced water. Frontiers in Microbiology. 10.3389/fmicb.2012.00175 PMC3349271 22590465

