# Supplementary figures and images for: Biogeochemical Typing of Paddy Field by a Data-Driven Approach Revealing Sub-Systems within a Complex Environment - A Pipeline to Filtrate, Organize and Frame Massive Dataset from Multi-Omics Analyses
Source: PLoS One. 2014 Oct 20;9(10):e110723. doi: 10.1371/journal.pone.0110723 (PMC4203823; doi:10.1371/journal.pone.0110723)

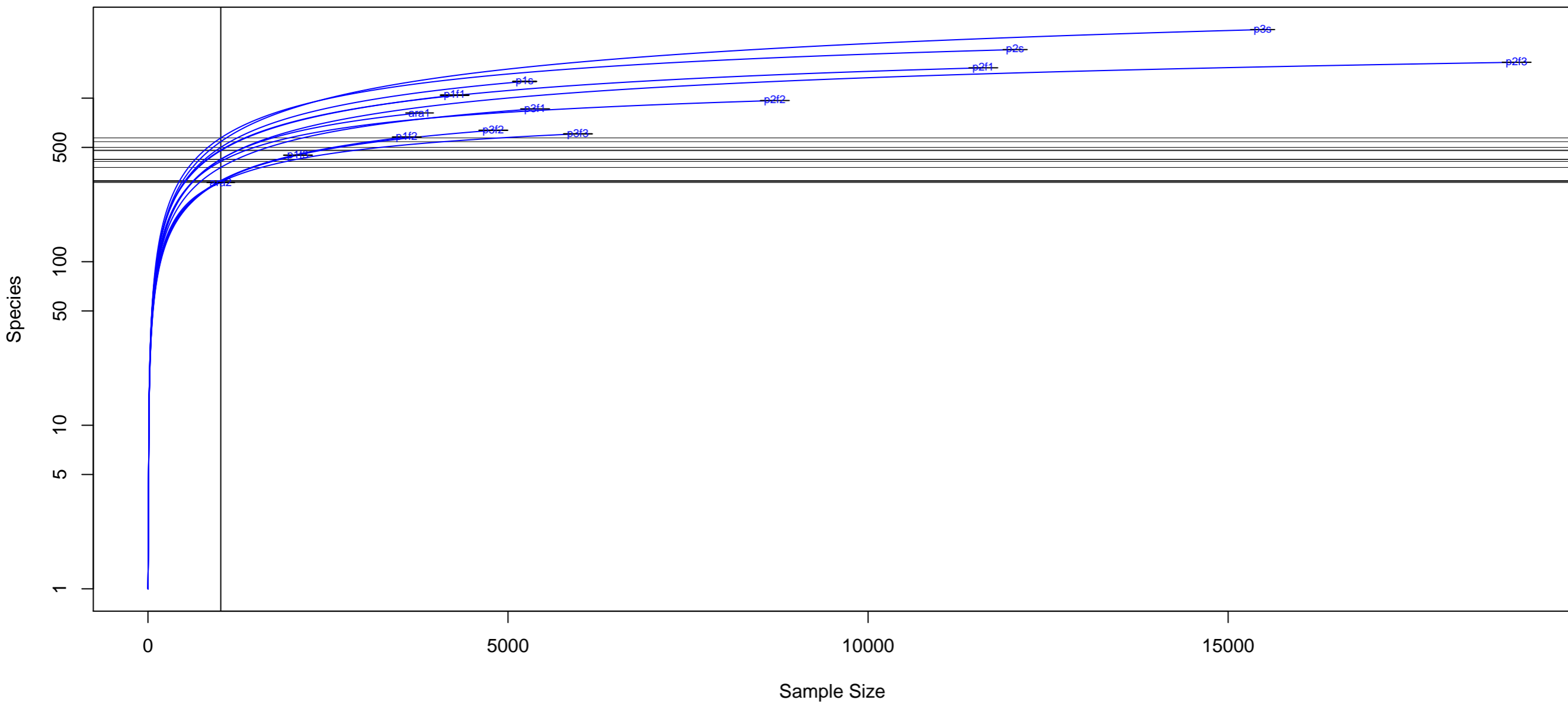

Supplement: Figure S1 — Rarefaction curve to total Archaea OTUs. Rarefaction curve for Archaea rRNA OTUs for all samples. X-axis: number of readings. Y-axis: number of species (log). (PDF) [file pone.0110723.s001.pdf]

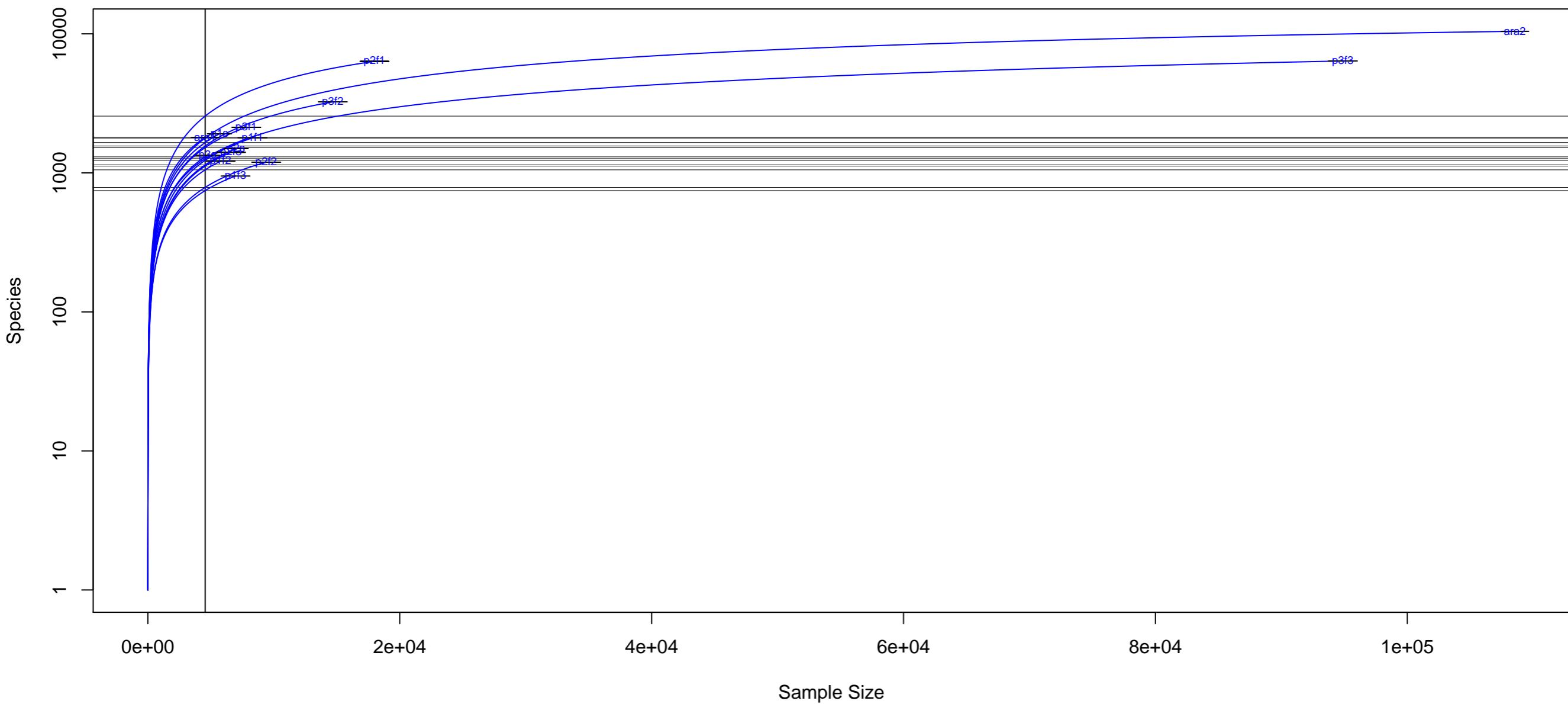

Supplement: Figure S2 — Rarefaction curve to toatal 16S rRNA OTUs. Rarefaction curve for 16S rRNA OTUs for all samples. X-axis: number of readings. Y-axis: number of species (log). (PDF) [file pone.0110723.s002.pdf]

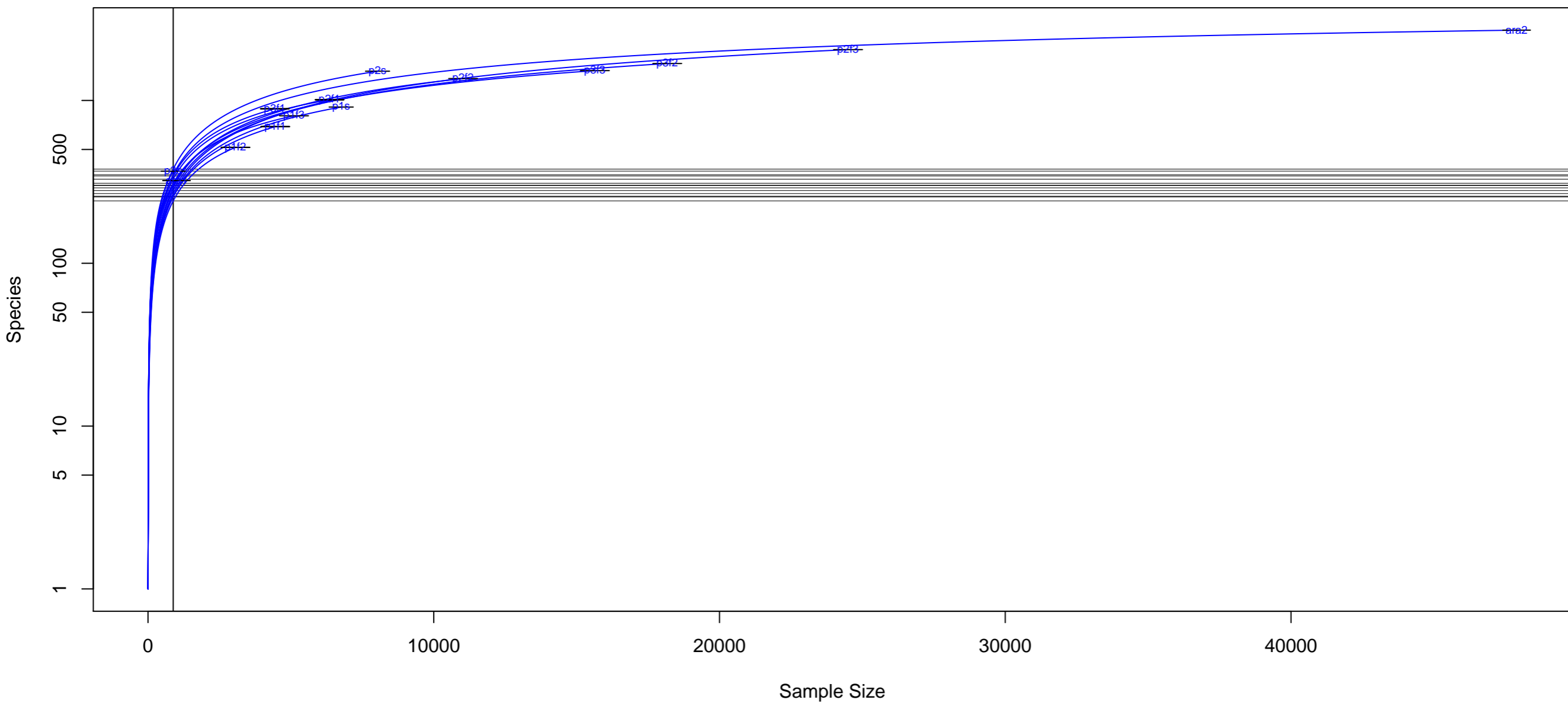

Supplement: Figure S3 — Rarefaction curve to total 18S rRNA OTUs. Rarefaction curve for 18S rRNA OTUs for all samples. X-axis: number of readings. Y-axis: number of species (log). (PDF) [file pone.0110723.s003.pdf]

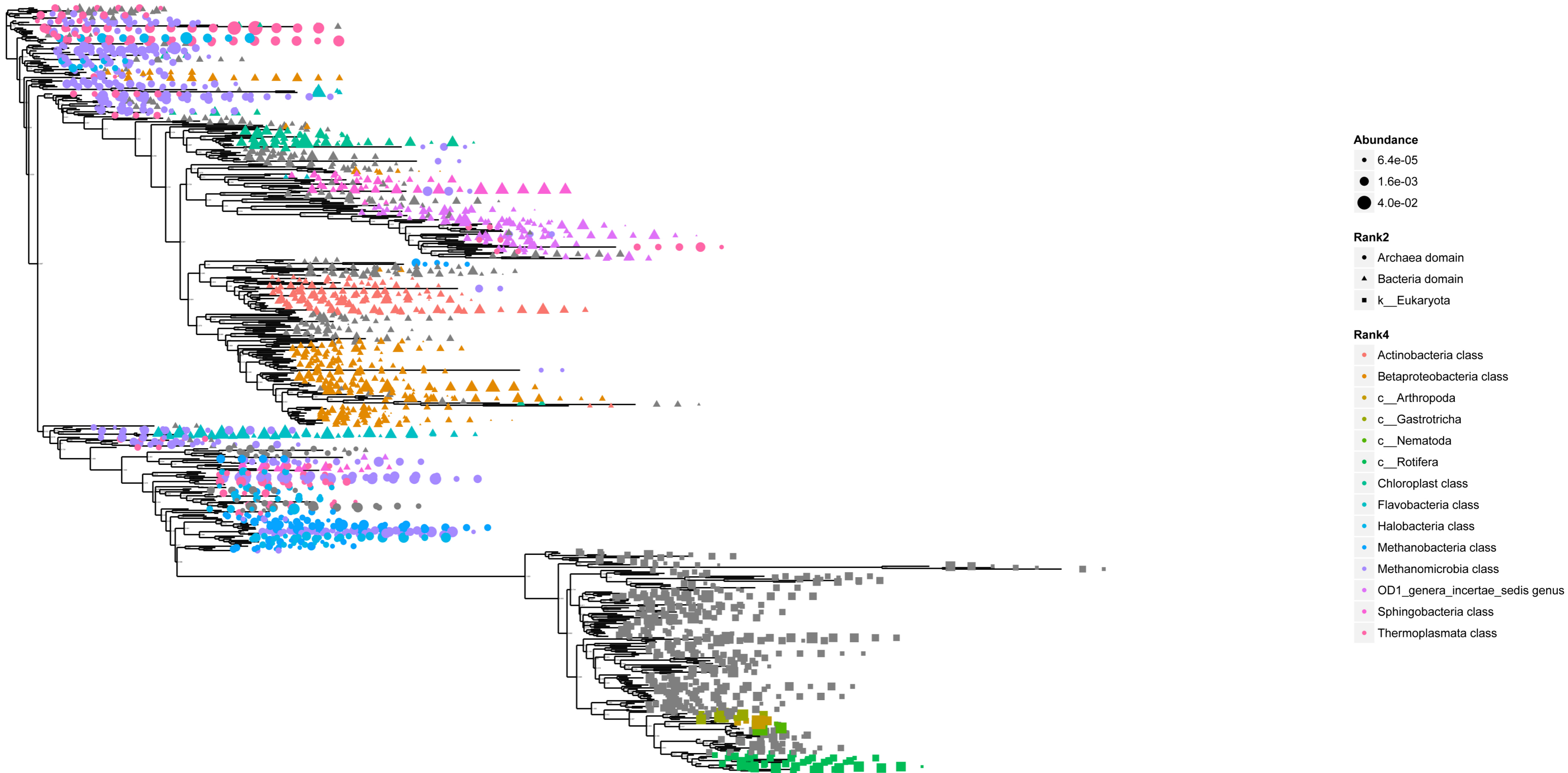

Supplement: Figure S4 — Emulation of a phylogenetic tree for BGC type I. Abundance range indicated by the size of the symbol indicated at upper right. Symbols indicative of the domain indicated at middle right. Colour codes for the most abundant classes indicated at lower right. The number of symbols for each branch is related to the number of sampling points within the OTUs present. (PDF) [file pone.0110723.s004.pdf]

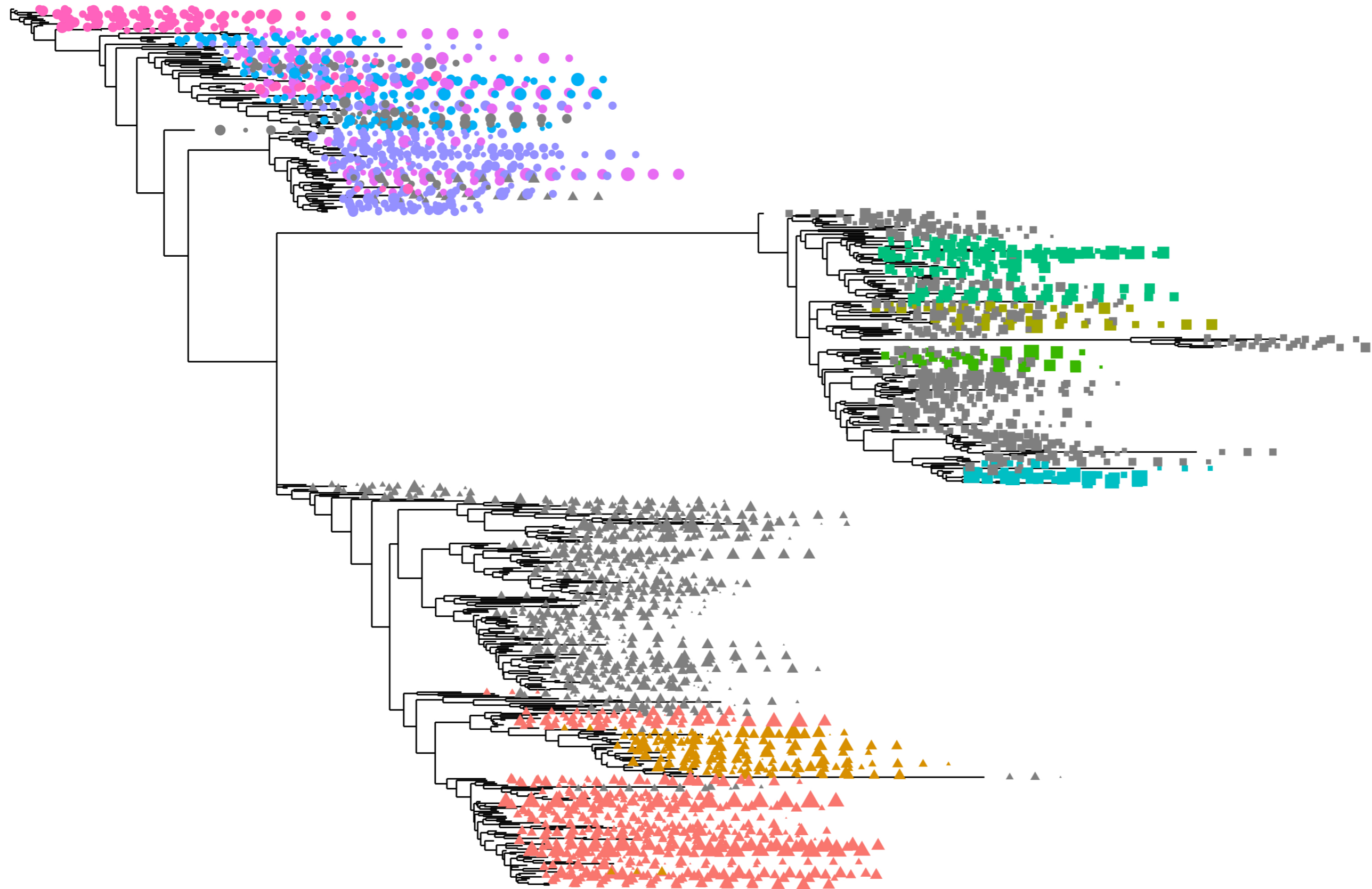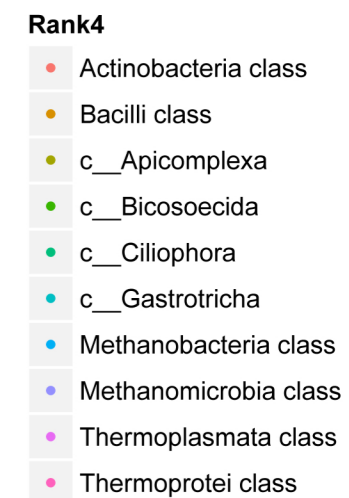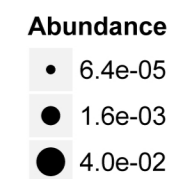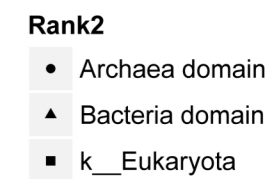

Supplement: Figure S5 — Emulation of a phylogenetic tree for BGC type II. Abundance range indicated by the size of the symbol indicated at upper right. Symbols indicative of the domain indicated at middle right. Colour codes for the most abundant classes indicated at lower right. The number of symbols for each branch is related to the number of sampling points within the OTUs present. (PDF) [file pone.0110723.s005.pdf]

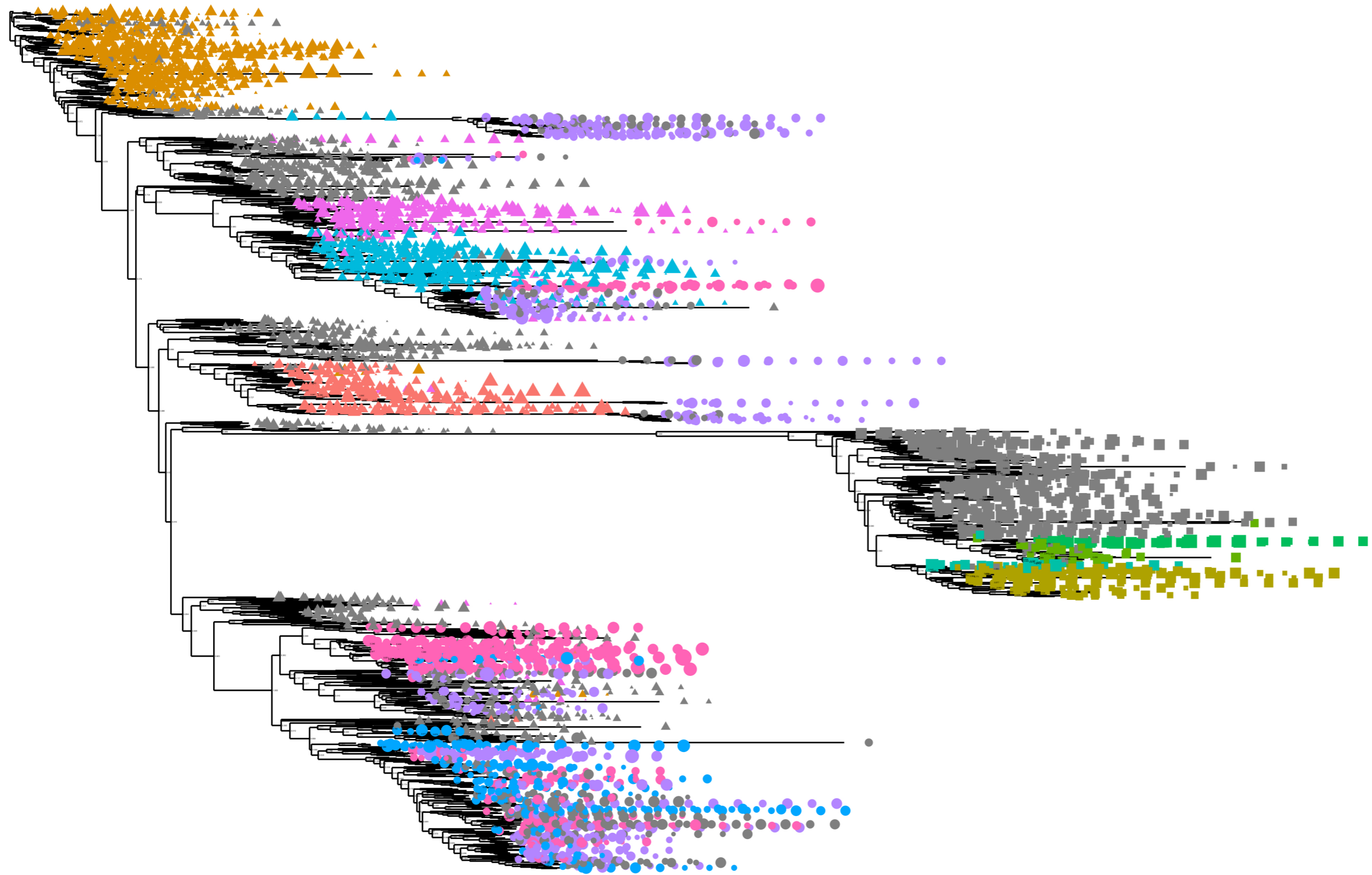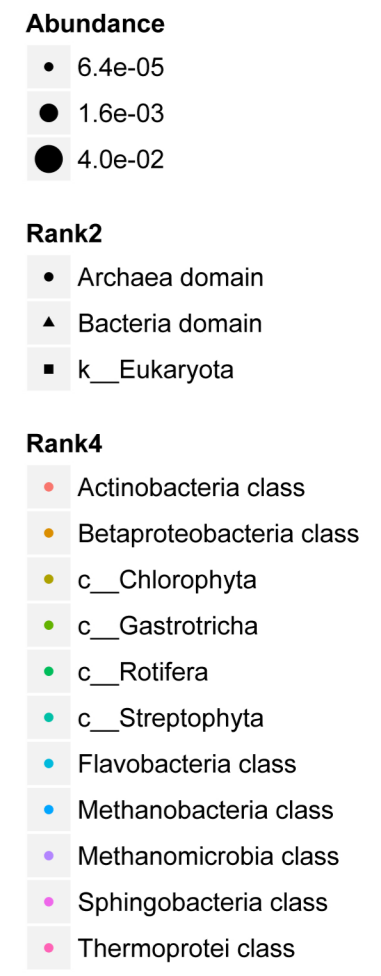

Supplement: Figure S6 — Emulation of a phylogenetic tree for BGC type III. Abundance range indicated by the size of the symbol indicated at upper right. Symbols indicative of the domain indicated at middle right. Colour codes for the most abundant classes indicated at lower right. The number of symbols for each branch is related to the number of sampling points within the OTUs present. (PDF) [file pone.0110723.s006.pdf]

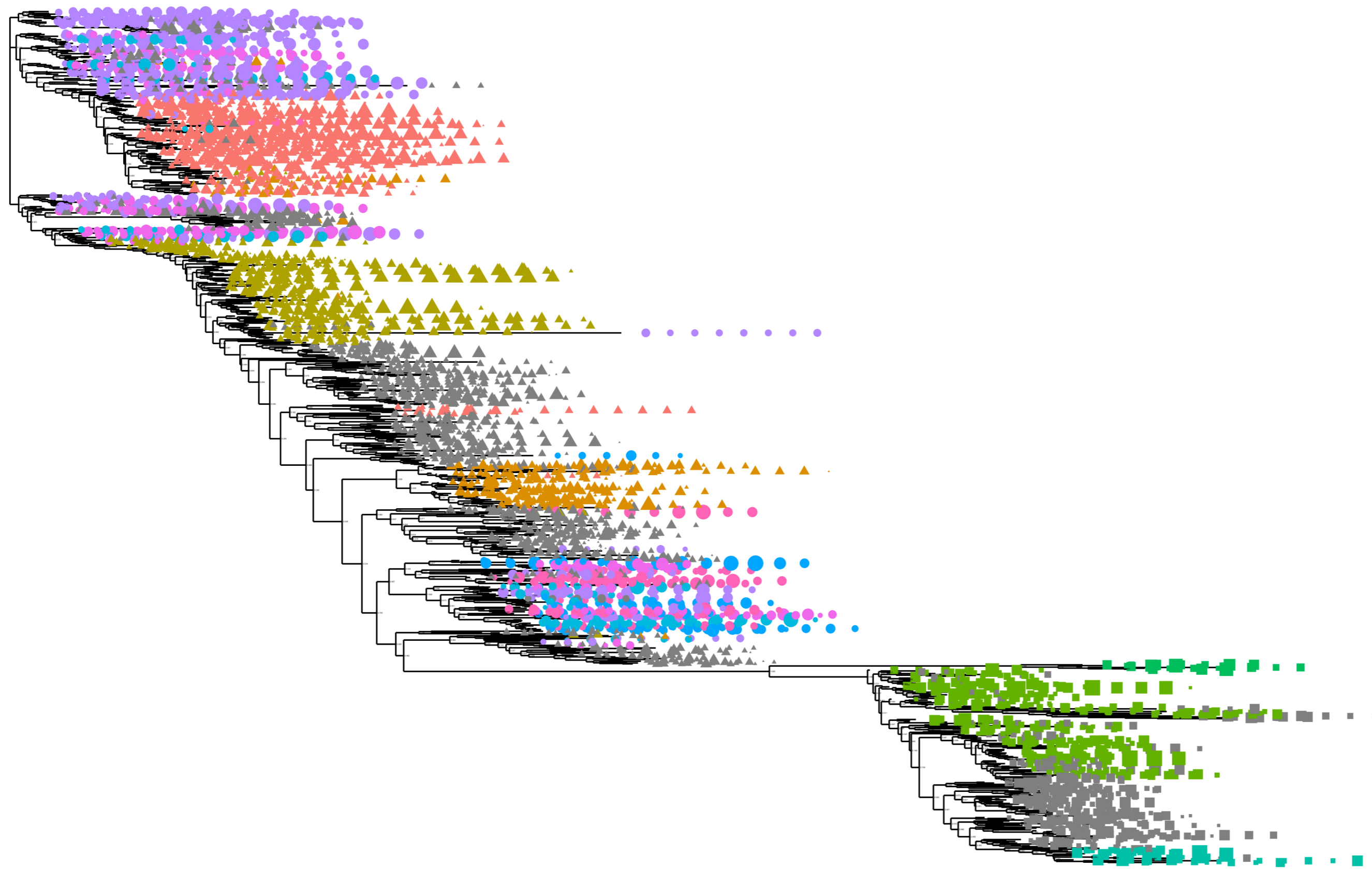

Supplement: Figure S7 — Emulation of a phylogenetic tree for BGC type IV. Abundance range indicated by the size of the symbol indicated at upper right. Symbols indicative of the domain indicated at middle right. Colour codes for the most abundant classes indicated at lower right. The number of symbols for each branch is related to the number of sampling points within the OTUs present. (PDF) [file pone.0110723.s007.pdf]

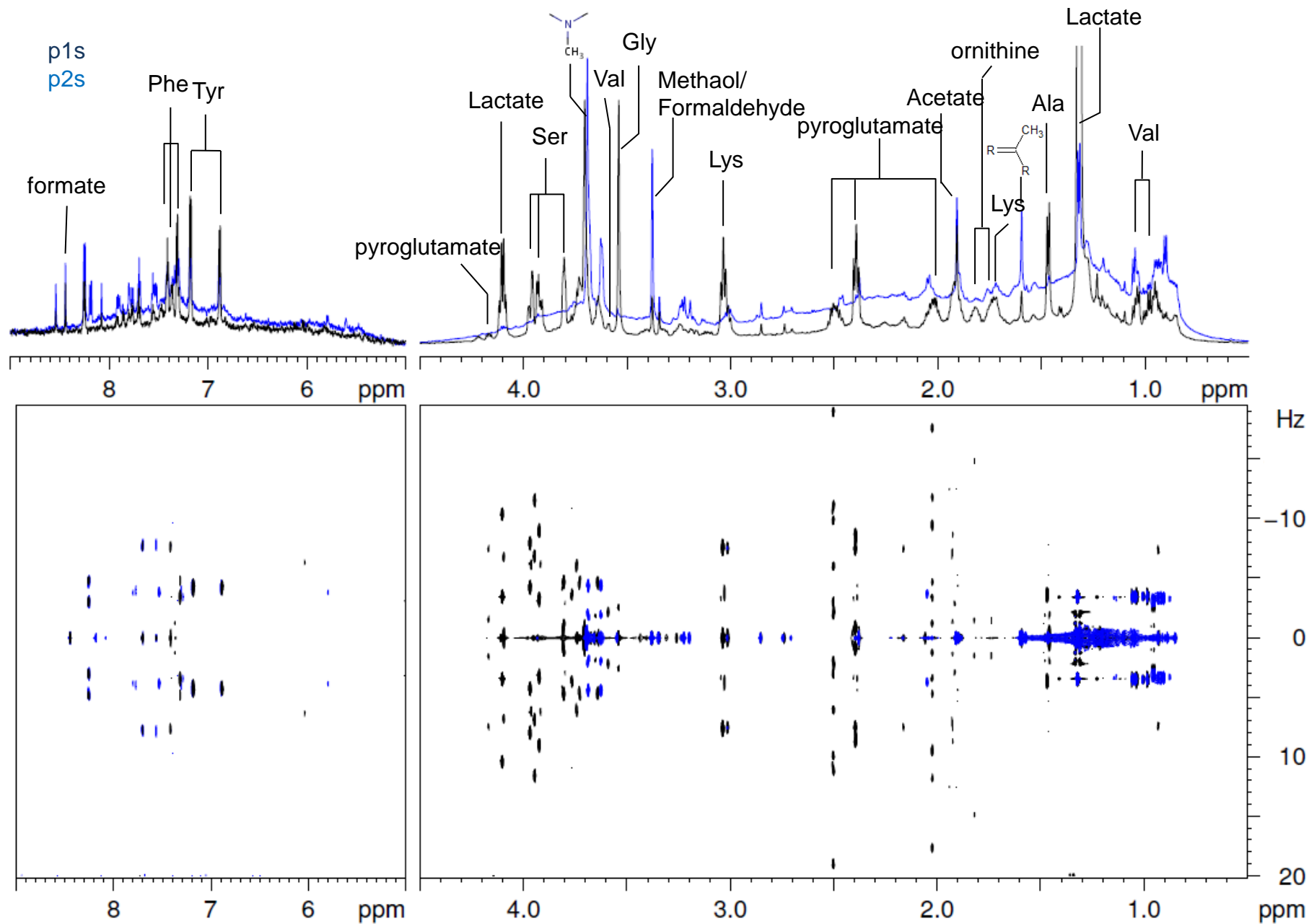

Supplement: Figure S8 — J-resolved 1H-NMR with annotations. J-resolved 1H-NMR analysis for aromatic and non-aromatic regions of the spectra with annotations. (PDF) [file pone.0110723.s008.pdf]

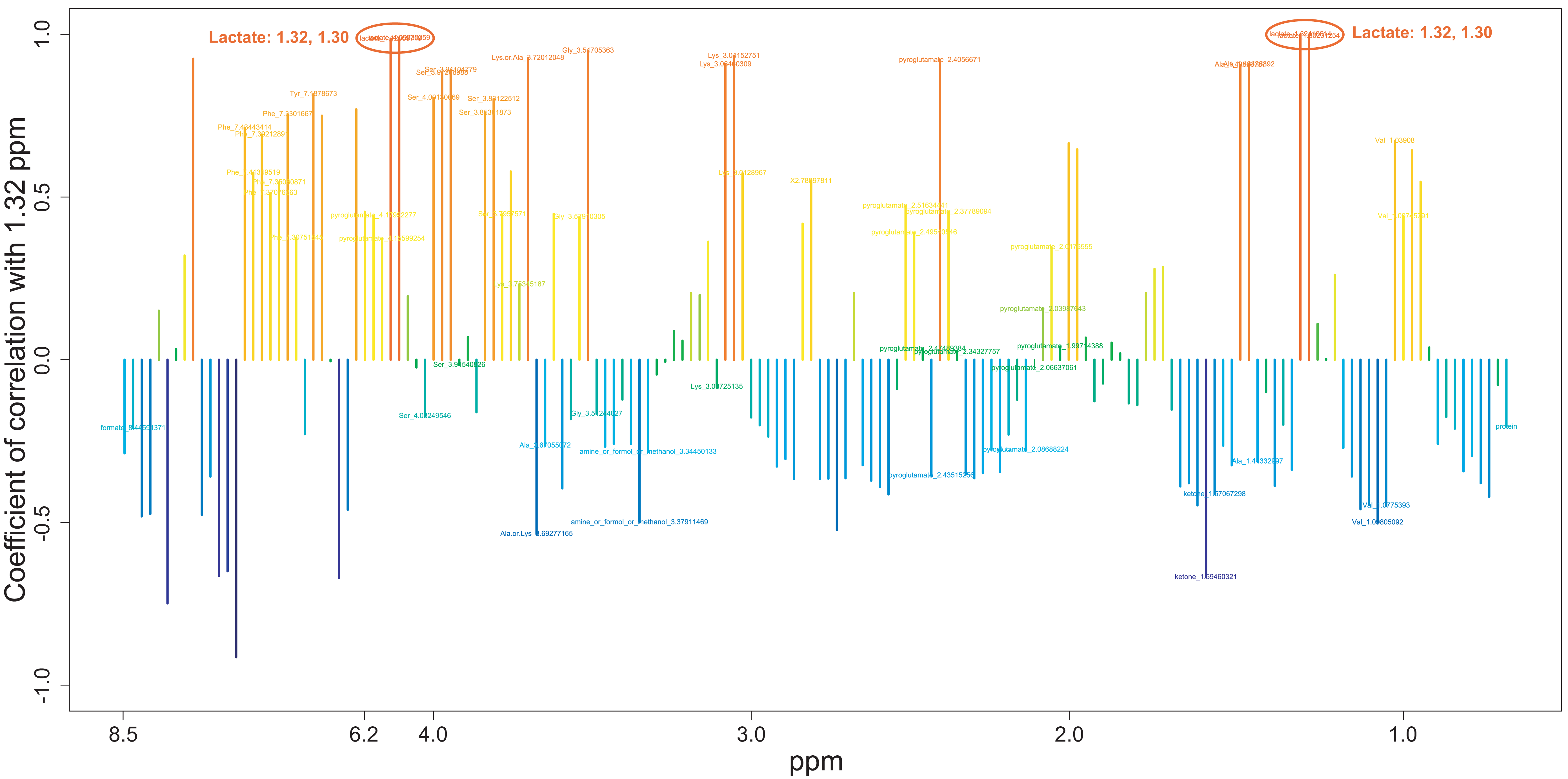

Supplement: Figure S9 — 1D-STOCSY correlation for chemical shift 1.32 ppm. 1D-STOCSY with centroid at 1.32 ppm showing high correlation with the other chemical shifts from the lactate assignment. X-axis: chemical shifts. Y-axis: degree of correlation. Colours simplify visualization: cold colours indicate negative correlations and hot colours indicate positive correlations. (TIF) [file pone.0110723.s009.tif]

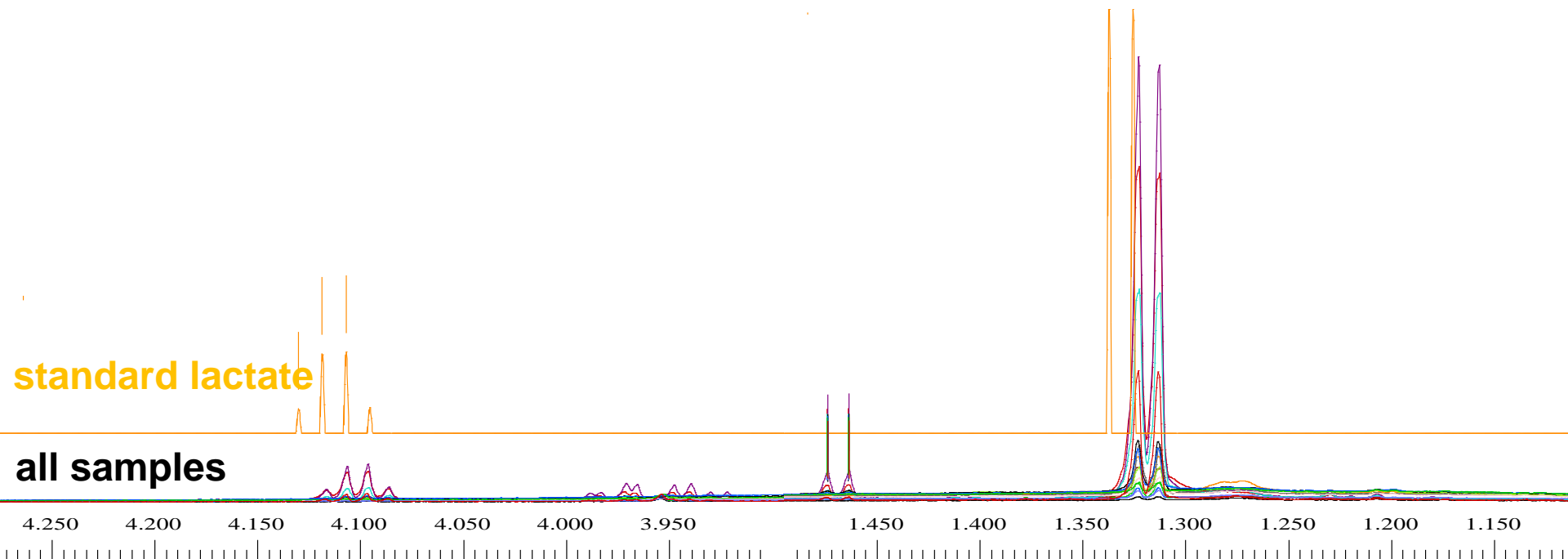

$^1\text{H}$ -NMR (ppm)

Supplement: Figure S10 — Comparison between the 1H-NMR spectra against the Bruker AMIX database for the pure compound lactate (lactic acid). The 1HNMR spectra with lower baselines are from this study. The upward-shifted baseline spectrum in orange is the lactate spectrum provided in Bruker AMIX software. Numbers indicate chemical shift. (PDF) [file pone.0110723.s010.pdf]

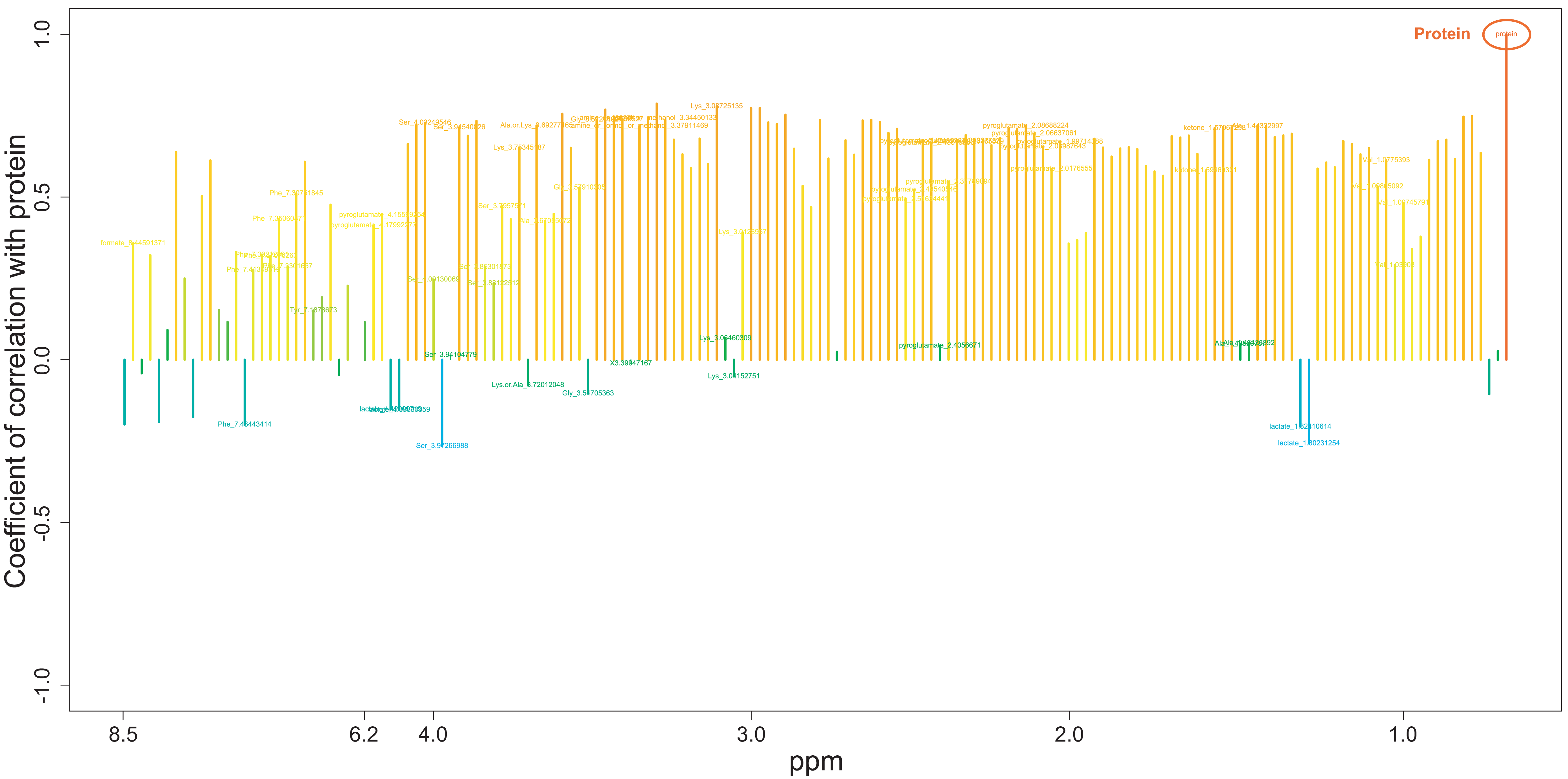

Supplement: Figure S11 — 1D-STOCSY correlation for the integration annotated as protein. 1D-STOCSY with centroid at the region of interest integrated and annotated as protein showing high correlation with most of the chemical shifts from the spectra. X-axis: chemical shifts. Y-axis: degree of correlation. Colours simplify visualization: cold colours indicate negative correlations and hot colours indicate positive correlations. (TIF) [file pone.0110723.s011.tif]
