# Supplementary material for: Biogeochemical Typing of Paddy Field by a Data-Driven Approach Revealing Sub-Systems within a Complex Environment - A Pipeline to Filtrate, Organize and Frame Massive Dataset from Multi-Omics Analyses
Source: PLoS One. 2014 Oct 20;9(10):e110723. doi: 10.1371/journal.pone.0110723 (PMC4203823; doi:10.1371/journal.pone.0110723)
Supplement: Figure S12 — Percentage of Archaea OTUs for BGC type I. Archaeal OTUs for BGC type I collapsed to the class level or beyond according to the next divergence on the taxon presented. The four most abundant taxa are shown, with others collapsed. (PDF) [file pone.0110723.s012.pdf]

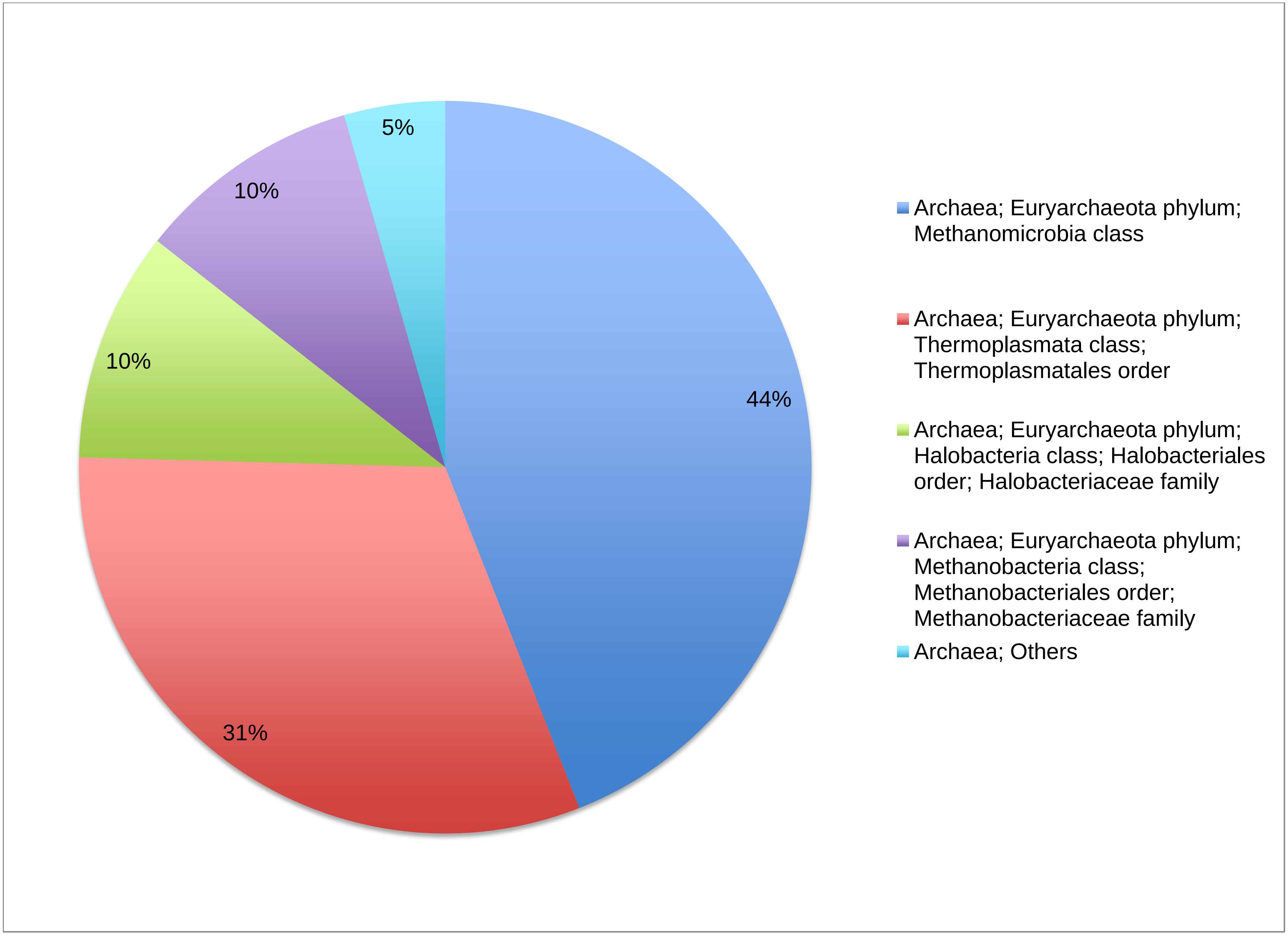
5%
10%
10%
44%
31%
Archaea; Euryarchaeota phylum; Methanomicrobia class
Archaea; Euryarchaeota phylum; Thermoplasmata class; Thermoplasmatales order
Archaea; Euryarchaeota phylum; Halobacteria class; Halobacteriales order; Halobacteriaceae family
Archaea; Euryarchaeota phylum; Methanobacteria class; Methanobacteriales order; Methanobacteriaceae family
Archaea; Others
