# Supplementary material for: Biogeochemical Typing of Paddy Field by a Data-Driven Approach Revealing Sub-Systems within a Complex Environment - A Pipeline to Filtrate, Organize and Frame Massive Dataset from Multi-Omics Analyses
Source: PLoS One. 2014 Oct 20;9(10):e110723. doi: 10.1371/journal.pone.0110723 (PMC4203823; doi:10.1371/journal.pone.0110723)
Supplement: Figure S13 — Percentage of Archaea OTUs for BGC type II. Archaeal OTUs for BGC type II collapsed to the class level or beyond according to the next divergence on the taxon presented. The four most abundant taxa are shown, with others collapsed. (PDF) [file pone.0110723.s013.pdf]

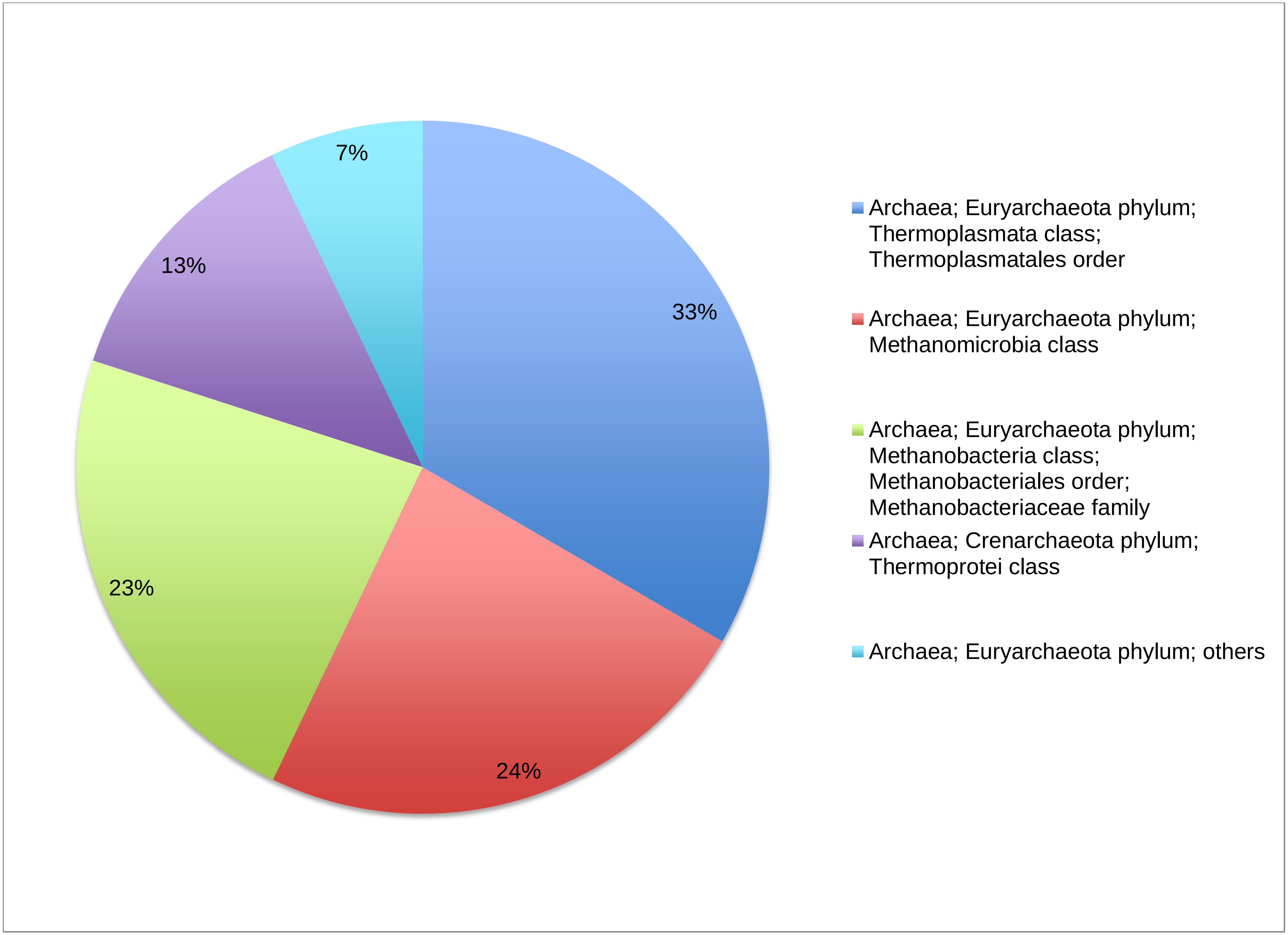
7%
33%
13%
23%
24%
Archaea; Euryarchaeota phylum; Thermoplasmata class; Thermoplasmatales order
Archaea; Euryarchaeota phylum; Methanomicrobia class
Archaea; Euryarchaeota phylum; Methanobacteria class; Methanobacteriales order; Methanobacteriaceae family
Archaea; Crenarchaeota phylum; Thermoprotei class
Archaea; Euryarchaeota phylum; others
