# Supplementary material for: Biogeochemical Typing of Paddy Field by a Data-Driven Approach Revealing Sub-Systems within a Complex Environment - A Pipeline to Filtrate, Organize and Frame Massive Dataset from Multi-Omics Analyses
Source: PLoS One. 2014 Oct 20;9(10):e110723. doi: 10.1371/journal.pone.0110723 (PMC4203823; doi:10.1371/journal.pone.0110723)
Supplement: Figure S14 — Percentage of Archaea OTUs for BGC type III. Archaeal OTUs for BGC type III collapsed to the class level or beyond according to the next divergence on the taxon presented. The four most abundant taxa are shown, with others collapsed. (PDF) [file pone.0110723.s014.pdf]

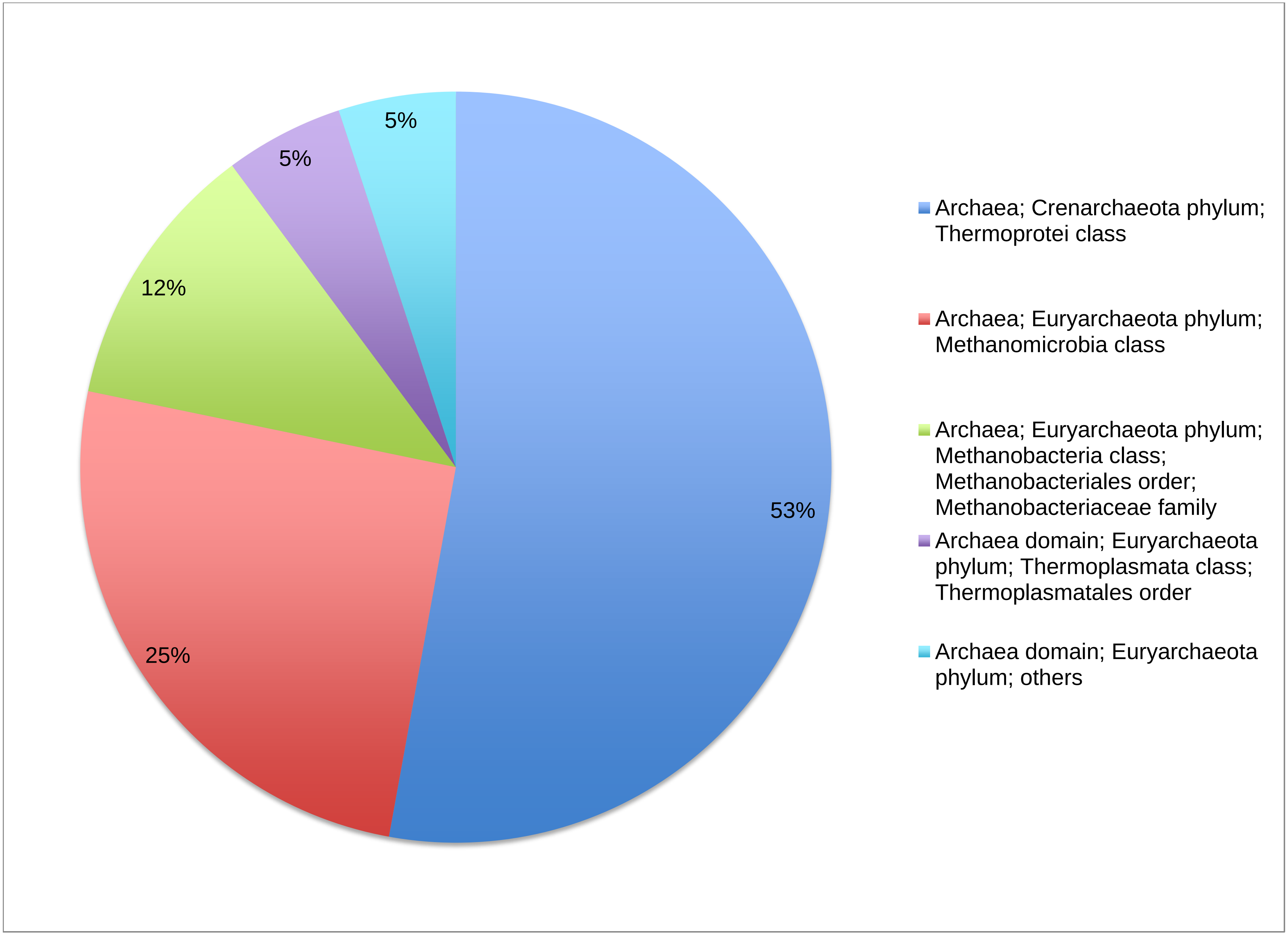

5%
5%
12%
53%
25%
Archaea; Crenarchaeota phylum; Thermoprotei class
Archaea; Euryarchaeota phylum; Methanomicrobia class
Archaea; Euryarchaeota phylum; Methanobacteria class; Methanobacteriales order; Methanobacteriaceae family
Archaea domain; Euryarchaeota phylum; Thermoplasmata class; Thermoplasmatales order
Archaea domain; Euryarchaeota phylum; others
