# Supplementary material for: Biogeochemical Typing of Paddy Field by a Data-Driven Approach Revealing Sub-Systems within a Complex Environment - A Pipeline to Filtrate, Organize and Frame Massive Dataset from Multi-Omics Analyses
Source: PLoS One. 2014 Oct 20;9(10):e110723. doi: 10.1371/journal.pone.0110723 (PMC4203823; doi:10.1371/journal.pone.0110723)
Supplement: Figure S16 — Percentage of 16S rRNA OTUs for BGC type I. 16S OTUs for BGC type I collapsed to the class level or beyond according to the next divergence on the taxon presented. The four most abundant taxa are shown, with others collapsed. (PDF) [file pone.0110723.s016.pdf]

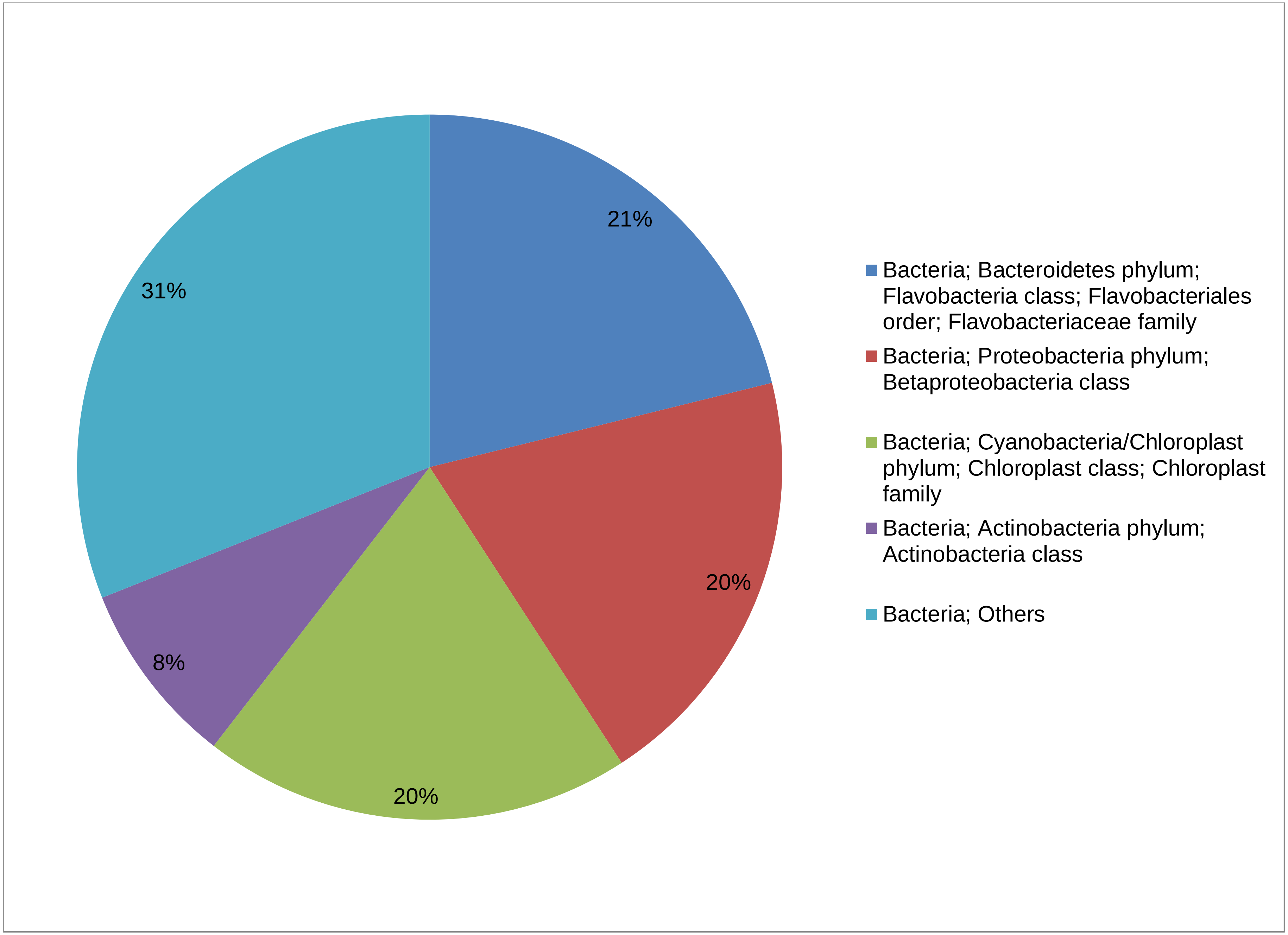

21%
20%
20%
8%
31%
Bacteria; Bacteroidetes phylum; Flavobacteria class; Flavobacteriales order; Flavobacteriaceae family
Bacteria; Proteobacteria phylum; Betaproteobacteria class
Bacteria; Cyanobacteria/Chloroplast phylum; Chloroplast class; Chloroplast family
Bacteria; Actinobacteria phylum; Actinobacteria class
Bacteria; Others
