# Supplementary material for: Biogeochemical Typing of Paddy Field by a Data-Driven Approach Revealing Sub-Systems within a Complex Environment - A Pipeline to Filtrate, Organize and Frame Massive Dataset from Multi-Omics Analyses
Source: PLoS One. 2014 Oct 20;9(10):e110723. doi: 10.1371/journal.pone.0110723 (PMC4203823; doi:10.1371/journal.pone.0110723)
Supplement: Figure S17 — Percentage of 16S rRNA OTUs for BGC type II. 16S OTUs for BGC type II collapsed to the class level or beyond according to the next divergence on the taxon presented. The four most abundant taxa are shown, with others collapsed. (PDF) [file pone.0110723.s017.pdf]

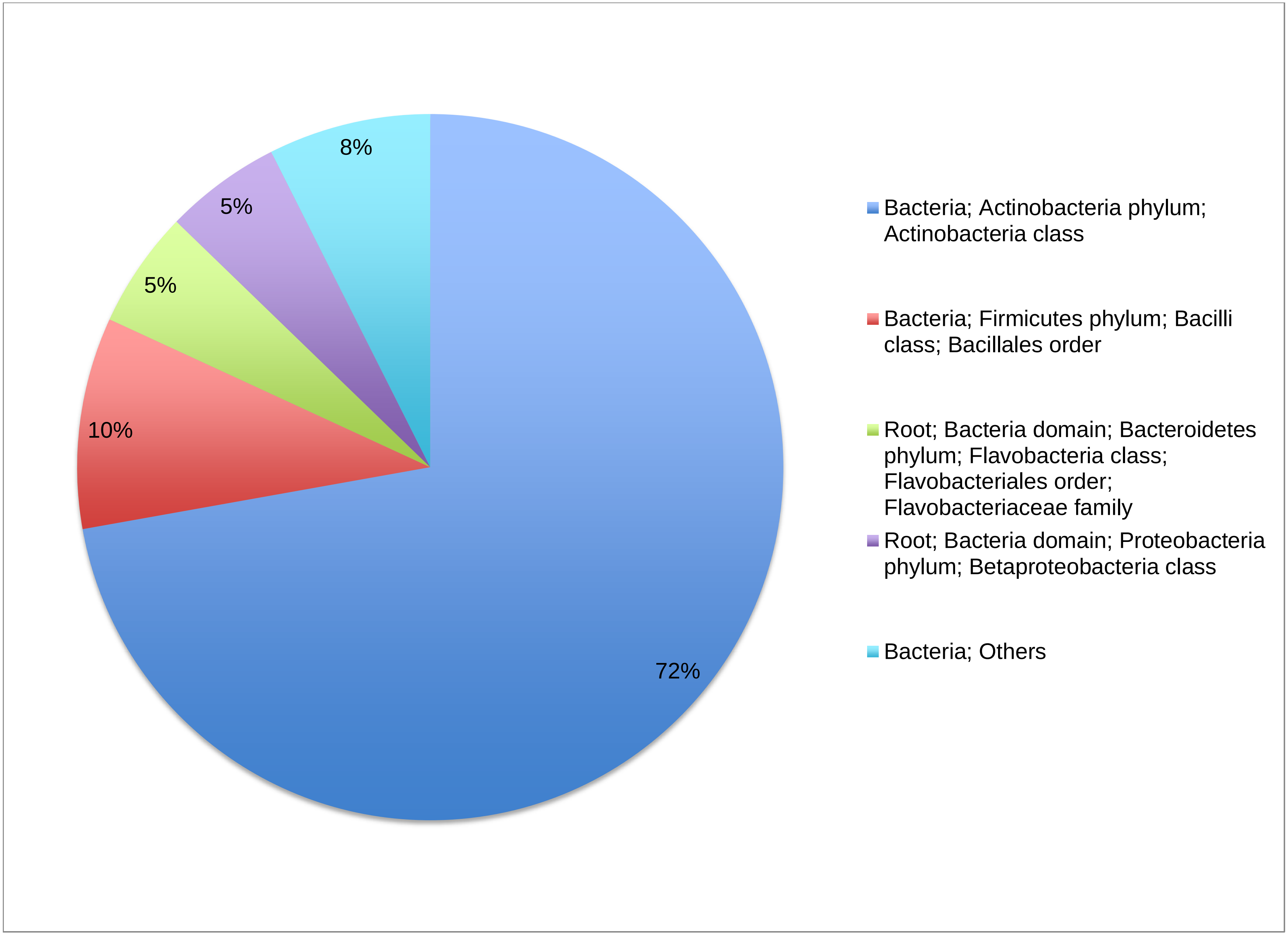
8%
5%
5%
10%
72%
Bacteria; Actinobacteria phylum; Actinobacteria class
Bacteria; Firmicutes phylum; Bacilli class; Bacillales order
Root; Bacteria domain; Bacteroidetes phylum; Flavobacteria class; Flavobacteriales order; Flavobacteriaceae family
Root; Bacteria domain; Proteobacteria phylum; Betaproteobacteria class
Bacteria; Others
