# Supplementary material for: Biogeochemical Typing of Paddy Field by a Data-Driven Approach Revealing Sub-Systems within a Complex Environment - A Pipeline to Filtrate, Organize and Frame Massive Dataset from Multi-Omics Analyses
Source: PLoS One. 2014 Oct 20;9(10):e110723. doi: 10.1371/journal.pone.0110723 (PMC4203823; doi:10.1371/journal.pone.0110723)
Supplement: Figure S18 — Percentage of 16S rRNA OTUs for BGC type III. 16S rRNA OTUs for BGC type III collapsed to the class level or beyond according to the next divergence on the taxon presented. The four most abundant taxa are shown, with others collapsed. (PDF) [file pone.0110723.s018.pdf]

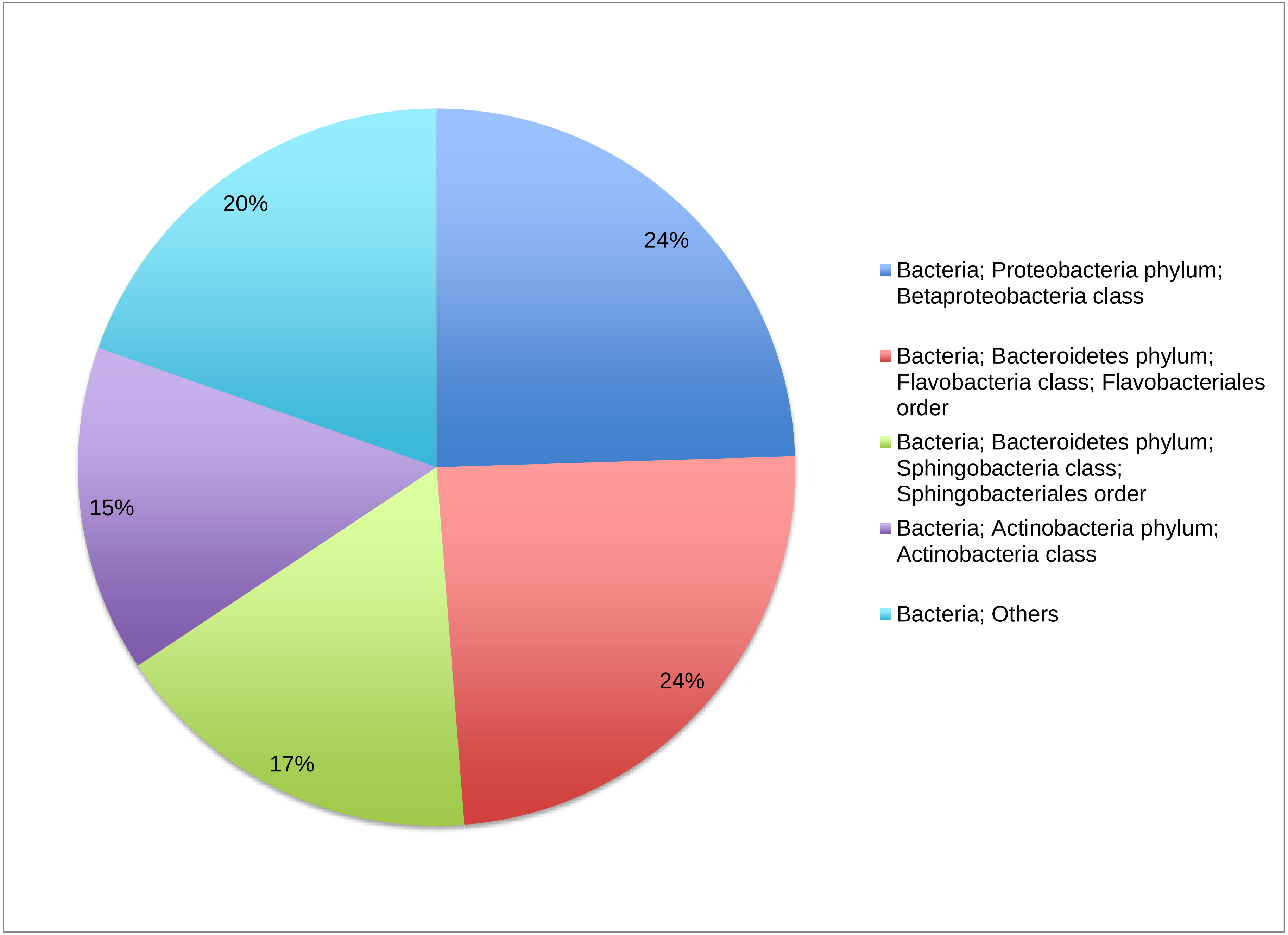
24%
24%
17%
15%
20%
Bacteria; Proteobacteria phylum; Betaproteobacteria class
Bacteria; Bacteroidetes phylum; Flavobacteria class; Flavobacteriales order
Bacteria; Bacteroidetes phylum; Sphingobacteria class; Sphingobacteriales order
Bacteria; Actinobacteria phylum; Actinobacteria class
Bacteria; Others
