# Supplementary material for: Biogeochemical Typing of Paddy Field by a Data-Driven Approach Revealing Sub-Systems within a Complex Environment - A Pipeline to Filtrate, Organize and Frame Massive Dataset from Multi-Omics Analyses
Source: PLoS One. 2014 Oct 20;9(10):e110723. doi: 10.1371/journal.pone.0110723 (PMC4203823; doi:10.1371/journal.pone.0110723)
Supplement: Figure S20 — Percentage of 18S rRNA OTUs for BGC type I. 18S OTUs for BGC type I collapsed to the class level or beyond according to the next divergence on the taxon presented. The four most abundant taxa are shown, with others collapsed. (PDF) [file pone.0110723.s020.pdf]

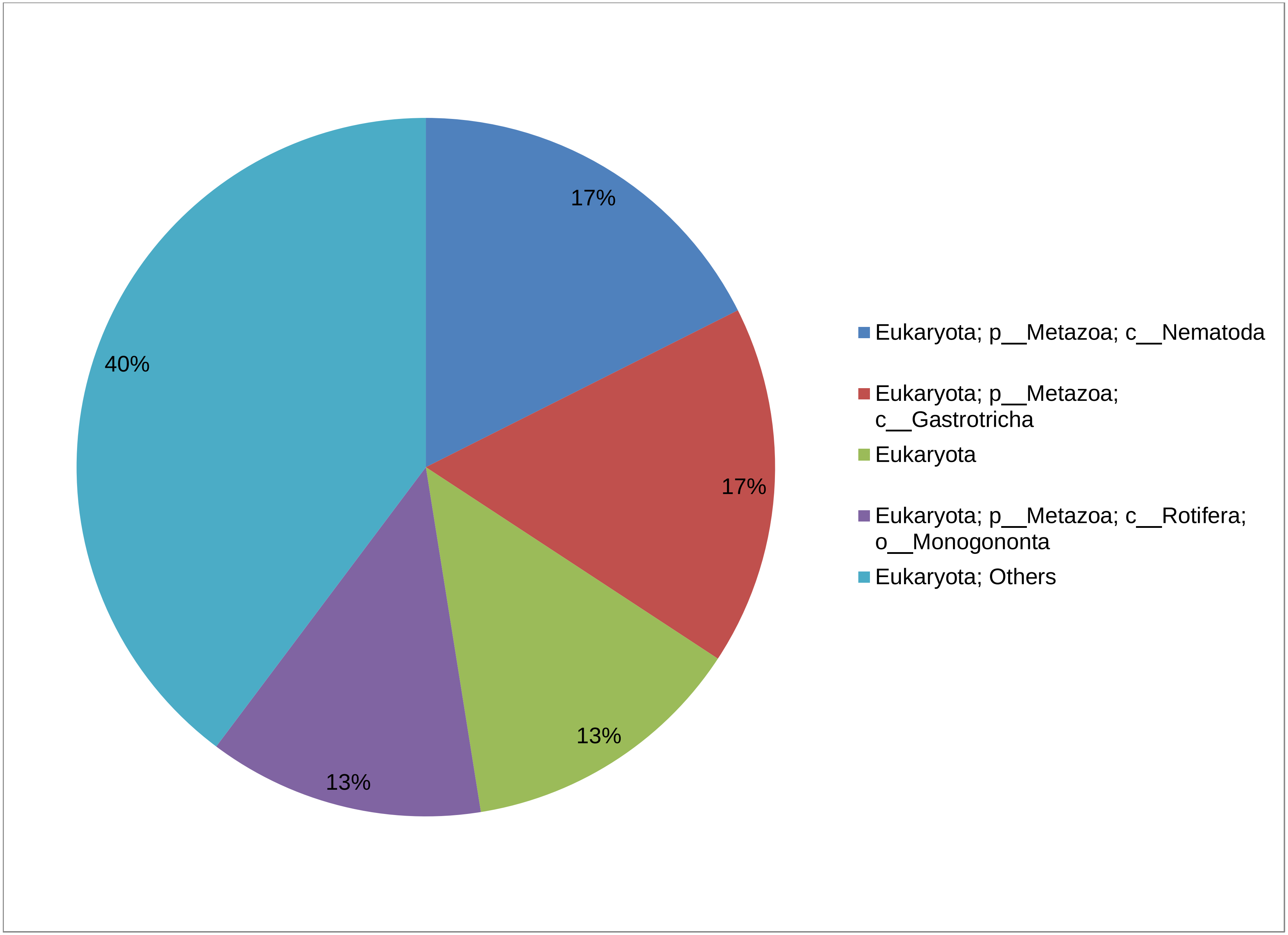
17%
17%
13%
13%
40%
Eukaryota; p__Metazoa; c__Nematoda
Eukaryota; p__Metazoa; c__Gastrotricha
Eukaryota
Eukaryota; p__Metazoa; c__Rotifera; o__Monogononta
Eukaryota; Others
