# Supplementary material for: Biogeochemical Typing of Paddy Field by a Data-Driven Approach Revealing Sub-Systems within a Complex Environment - A Pipeline to Filtrate, Organize and Frame Massive Dataset from Multi-Omics Analyses
Source: PLoS One. 2014 Oct 20;9(10):e110723. doi: 10.1371/journal.pone.0110723 (PMC4203823; doi:10.1371/journal.pone.0110723)
Supplement: Figure S21 — Percentage of 18S rRNA OTUs for BGC type II. 18S OTUs for BGC type II collapsed to the class level or beyond according to the next divergence on the taxon presented. The four most abundant taxa are shown, with others collapsed. (PDF) [file pone.0110723.s021.pdf]

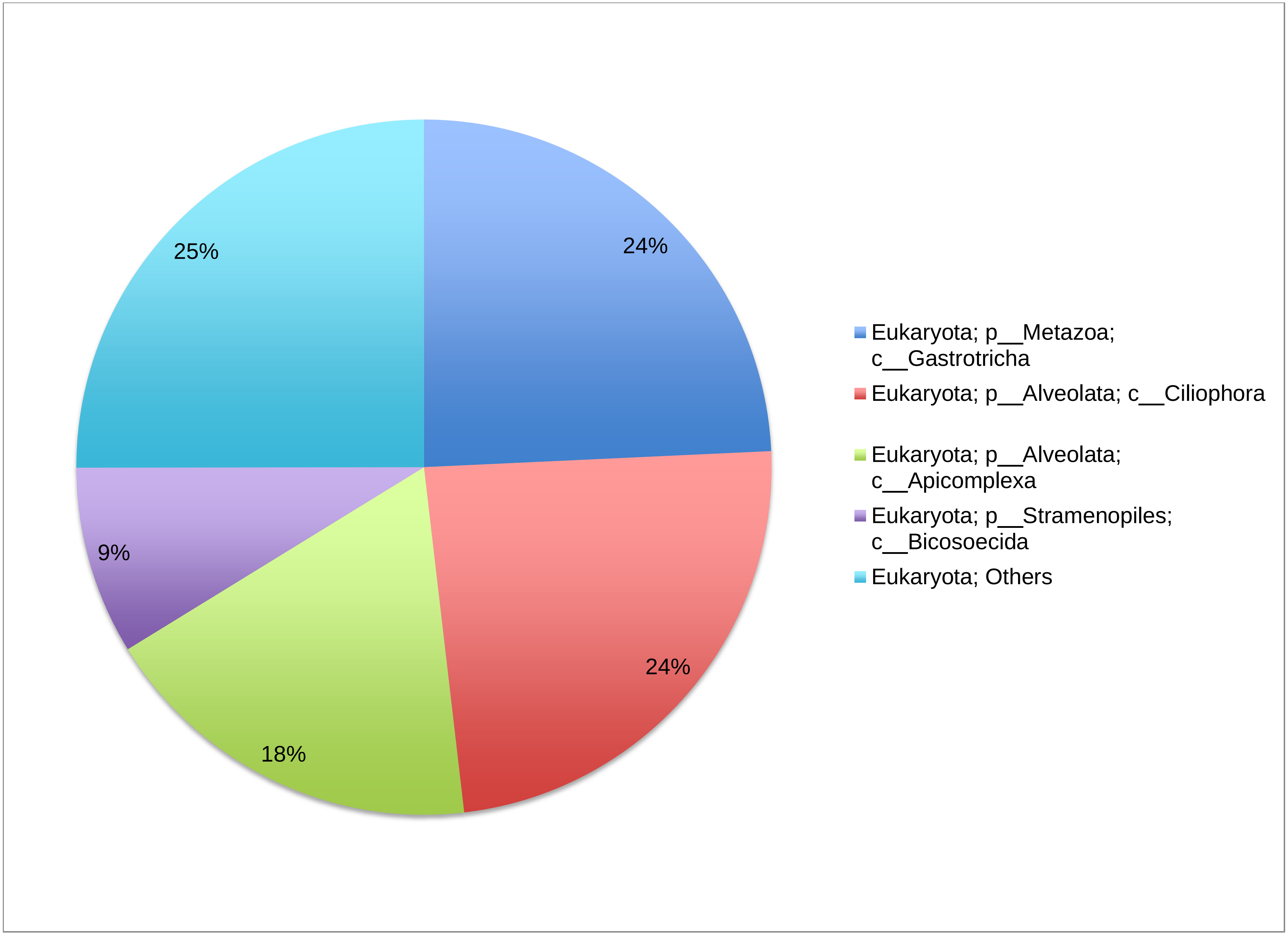

24%
24%
18%
9%
25%
Eukaryota; p__Metazoa; c__Gastrotricha
Eukaryota; p__Alveolata; c__Ciliophora
Eukaryota; p__Alveolata; c__Apicomplexa
Eukaryota; p__Stramenopiles; c__Bicosoecida
Eukaryota; Others
