# Supplementary material for: Biogeochemical Typing of Paddy Field by a Data-Driven Approach Revealing Sub-Systems within a Complex Environment - A Pipeline to Filtrate, Organize and Frame Massive Dataset from Multi-Omics Analyses
Source: PLoS One. 2014 Oct 20;9(10):e110723. doi: 10.1371/journal.pone.0110723 (PMC4203823; doi:10.1371/journal.pone.0110723)
Supplement: Figure S22 — Percentage of 18S rRNA OTUs for BGC type III. 18S rRNA OTUs for BGC type III collapsed to the class level or beyond according to the next divergence on the taxon presented. The four most abundant taxa are shown, with others collapsed. (PDF) [file pone.0110723.s022.pdf]

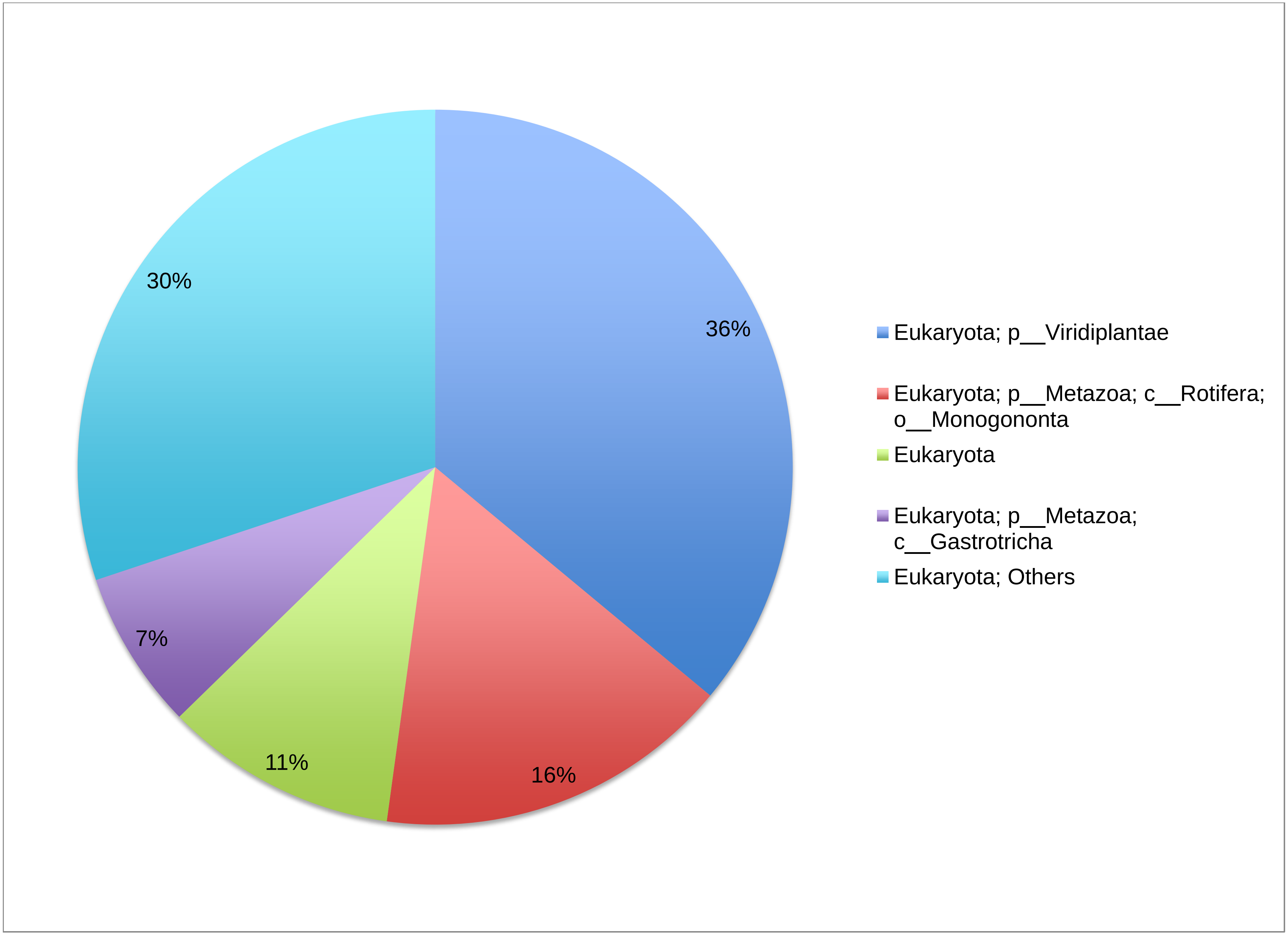
36%
16%
11%
7%
30%
Eukaryota; p__Viridiplantae
Eukaryota; p__Metazoa; c__Rotifera; o__Monogononta
Eukaryota
Eukaryota; p__Metazoa; c__Gastrotricha
Eukaryota; Others
