# Supplementary material for: Biogeochemical Typing of Paddy Field by a Data-Driven Approach Revealing Sub-Systems within a Complex Environment - A Pipeline to Filtrate, Organize and Frame Massive Dataset from Multi-Omics Analyses
Source: PLoS One. 2014 Oct 20;9(10):e110723. doi: 10.1371/journal.pone.0110723 (PMC4203823; doi:10.1371/journal.pone.0110723)
Supplement: Figure S23 — Percentage of 18S rRNA OTUs for BGC type IV. 18S rRNA OTUs for BGC type IV collapsed to the class level or beyond according to the next divergence on the taxon presented. The four most abundant taxa are shown, with others collapsed. (PDF) [file pone.0110723.s023.pdf]

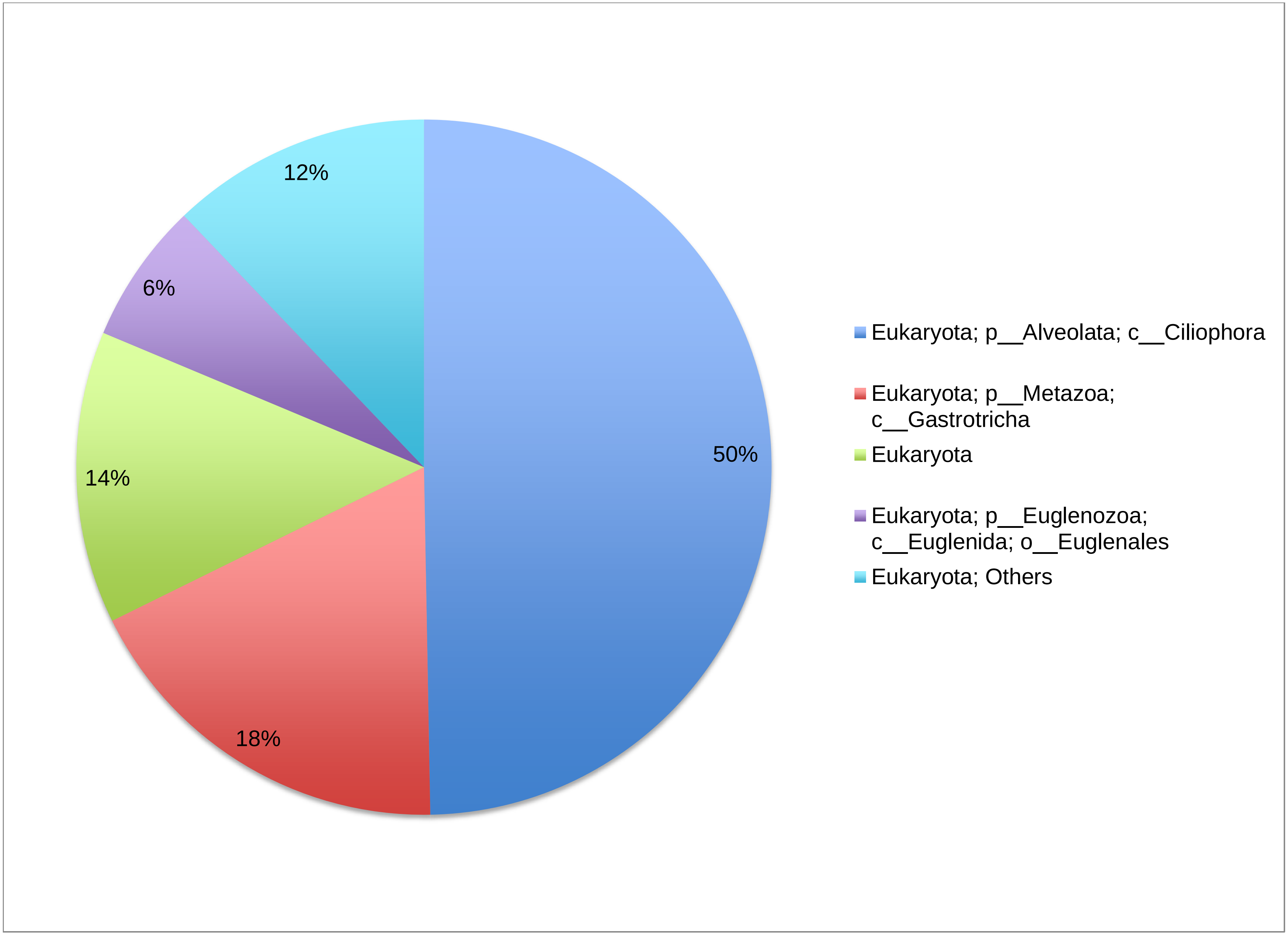
12%
6%
14%
18%
50%
Eukaryota; p__Alveolata; c__Ciliophora
Eukaryota; p__Metazoa; c__Gastrotricha
Eukaryota
Eukaryota; p__Euglenozoa; c__Euglenida; o__Euglenales
Eukaryota; Others
